# Conjugates of Aminoglycosides with Stapled Peptides as a Way to Target Antibiotic-Resistant Bacteria

**DOI:** 10.1021/acsomega.3c02071

**Published:** 2023-05-16

**Authors:** Julia Macyszyn, Michał Burmistrz, Adam Mieczkowski, Monika Wojciechowska, Joanna Trylska

**Affiliations:** † Centre of New Technologies, University of Warsaw, Banacha 2c, 02-097 Warsaw, Poland; ‡ Institute of Biochemistry and Biophysics, Polish Academy of Sciences, Pawinskiego 5a, 02-106 Warsaw, Poland

## Abstract

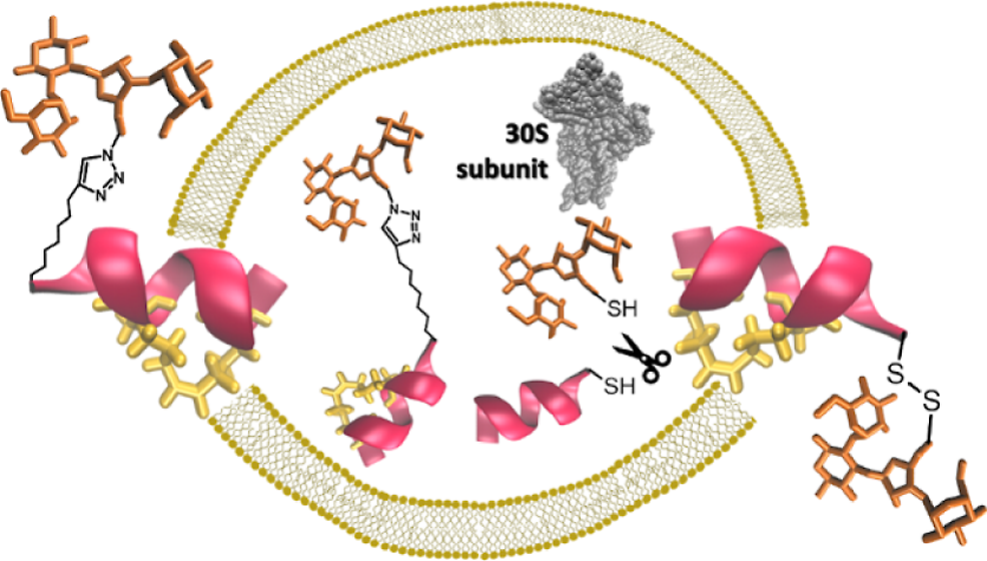

The misuse and overuse of antibiotics led to the development of bacterial resistance to existing aminoglycoside (AMG) antibiotics and limited their use. Consequently, there is a growing need to develop effective antimicrobials against multidrug-resistant bacteria. To target resistant strains, we propose to combine 2-deoxystreptamine AMGs, neomycin (NEO) and amikacin (AMK), with a membrane-active antimicrobial peptide anoplin and its hydrocarbon stapled derivative. The AMG–peptide hybrids were conjugated using the click chemistry reaction in solution to obtain a non-cleavable triazole linker and by disulfide bridge formation on the resin to obtain a linker cleavable in the bacterial cytoplasm. Homo-dimers connected via disulfide bridges between the N-terminus thiol analogues of anoplin and hydrocarbon stapled anoplin were also synthesized. These hybrid compounds show a notable increase in antibacterial and bactericidal activity, as compared to the unconjugated ones or their combinations, against Gram-positive and Gram-negative bacteria, especially for the strains resistant to AMK or NEO. The conjugates and disulfide peptide dimers exhibit low hemolytic activity on sheep red blood erythrocytes.

## Introduction

Tackling antibiotic resistance has become one of the world’s biggest challenges.^1^ Already 700,000 people die each year from drug-resistant bacterial infections. It is estimated that the death toll could rise to 10 million by 2050, which is more than today’s cancer death rate.^2^ In addition, because of the misuse of antibiotics during the COVID-19 epidemic, the problem exacerbated.^3,4^ Overuse of antibiotics to prevent or treat bacterial complications in infected patients has rapidly increased antibiotic resistance. Therefore, the development of new effective antimicrobial agents, especially against multidrug-resistant (MDR) strains, is essential.

Aminoglycosides (AMGs) are one of the first discovered broad-spectrum antibiotics active against Gram-positive and Gram-negative bacteria.^5,6^ Since World War II, they have been used in antibacterial therapy and medicinal chemistry.^7,8^ These positively charged, modified polysaccharides enter cells through pore channels via an energy-dependent mechanism.^9^ AMGs primarily target bacterial ribosomes and block protein translation, resulting in the inhibition of bacterial growth.^10−13^

However, bacteria developed several resistance mechanisms drastically reducing the effectiveness of AMG.^7,14,15^ These include enzymatic modifications of AMG by bacterial enzymes, active transport outside the bacterial cell by the efflux pumps, and mutations and methylations of ribosomal RNA.^7,16^ In efforts to overcome the resistance problem, AMGs have been chemically modified to increase their ribosomal RNA binding affinity, antibacterial activity and selectivity, and reduce their susceptibility to AMG-modifying enzymes.^16−20^ Apparently, AMG syntheses and modifications pose a challenge due to their structural complexity and rich stereochemistry.^10^

**Figure 1:**
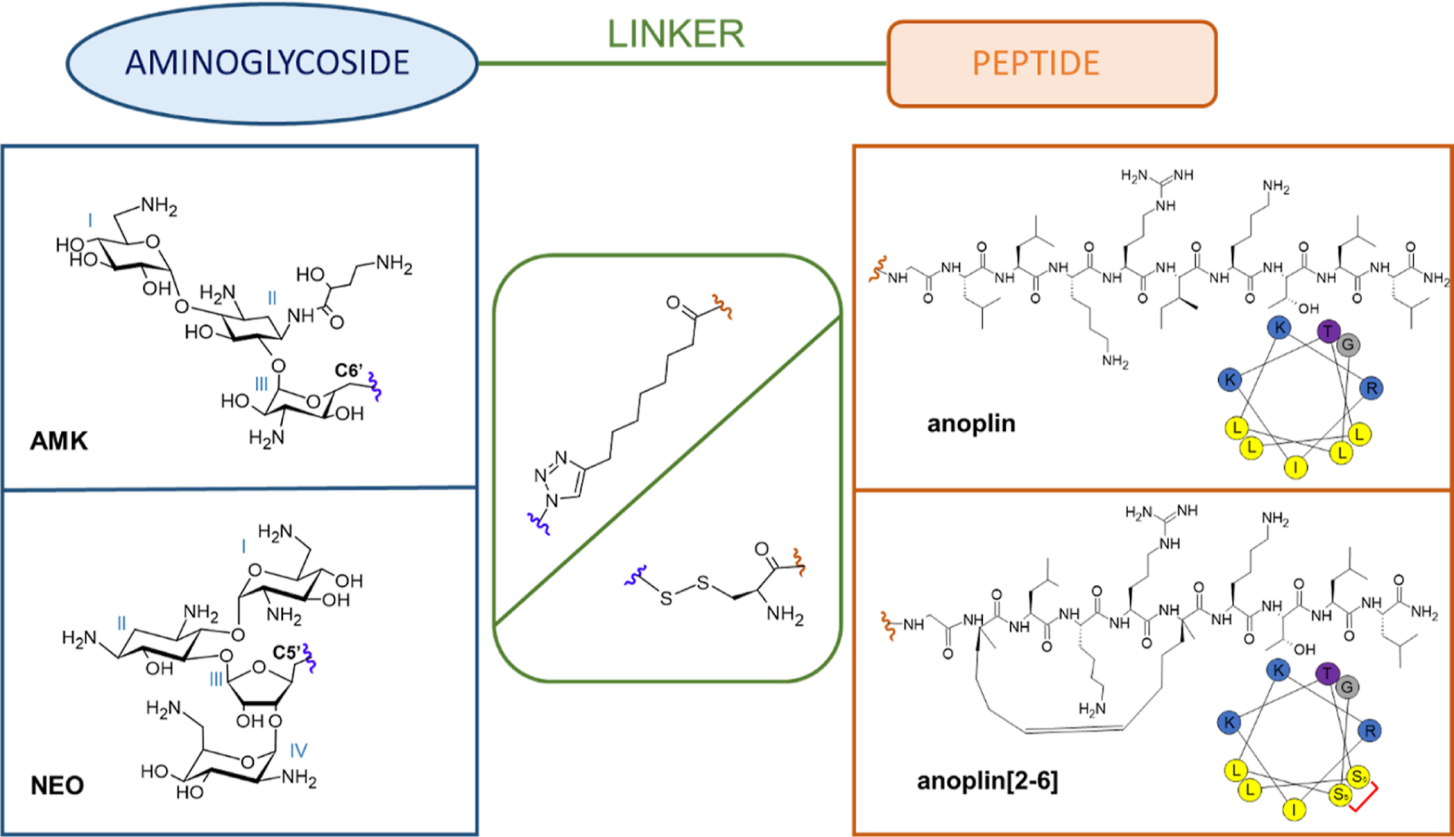
Chemical structures of the elements forming the conjugates: aminoglycosides [amikacin (AMK) and neomycin (NEO)], amphipathic peptides (anoplin and anoplin[2-6]), and the linker type (triazole and disulfide bond). The helical wheel projection (predicted by Heliquest^60^) of the peptides and the positions at which the elements are connected are also shown (red and blue waves).

Nevertheless, the modification of neomycin (NEO) in the C5′ position of ring III (Figure 1) by different functional groups has served as a viable strategy to address the resistance problem.^21−23^ These NEO modifications directed the development of new AMG.^24−26^ In addition, the primary hydroxyl group in the C6′ position of amikacin (AMK) was found suitable for the incorporation of hydrogen bond donors or acceptors and other functional groups (Figure 1).^27^ For example, AMK modifications by methylamine inserted in the C6′ position via the triazole ring showed a two-fold increase in activity against a resistant hospital-associated MRSA strain of *Staphylococcus aureus* ATCC 33591.

An important class of antibacterials are antimicrobial peptides (AMPs).^28−32^ Many AMPs, which adopt a helical conformation upon interaction with the bacterial membranes, have been discovered.^33^ The amphipathic nature of AMP allows them to selectively interact with the negatively charged bacterial cell surface and hydrophobic fatty acids.^29,33^ A common AMP antibacterial mechanism is associated with their ability to adopt an active secondary structure that permeabilizes and destabilizes the membrane.^34^ However, natural AMPs have many limitations, such as weak stability in the enzymatic environment and cytotoxicity.^34^ Therefore, many AMP modifications have been introduced to enhance their antibacterial activity and biostability and to decrease toxicity to eukaryotic cells.^35,36^

One of the methods used to modify peptides by initiating or stabilizing a helical structure is peptide stapling.^37^ The idea is based on replacing two amino acids located on the hydrophobic side of an amphipathic peptide with unnatural ones. These unnatural amino acids are inserted into the sequence between one turn of the helix or, in a longer peptide, two helical turns. Furthermore, a covalent bridge is formed between the side chains of the inserted amino acids.^37^ Therefore, hydrocarbon stapling can impart structural rigidity to the peptide and reinforce or improve the stability of the secondary structure (typically a helical conformation). This technique has become useful particularly for AMP.^38,39^ Several reports have shown that stapled antimicrobial peptides (StAMPs) adopt a stable helix, are resistant to proteases, destabilize bacterial membranes, and have better antibacterial activity.^40−43^ The therapeutic potential of such StAMP in vivo was also demonstrated.^42^

We focus on the anoplin peptide whose modifications and antibacterial potential have been recently studied.^44−50^ Anoplin is a naturally found amphipathic peptide (N_ter_-Gly-Leu-Leu-Lys-Arg-Ile-Lys-Thr-Leu-Leu-C_ter_) derived from the venom sac of the solitary wasp, with rather low antibacterial activity. However, we and others demonstrated the antibacterial potential of anoplin derivatives.^49,51^ Typically, amphipathic and stable secondary structures are key for cytoplasmic membrane disruption and effective antibacterial activity of peptides.^28^ We showed that anoplin adopts a helical structure in the presence of membrane mimics and lipopolysaccharides.^48^ We also showed that hydrocarbon stapling of anoplin stabilizes its helical structure and increases its proteolytic stability and antibacterial activity (up to 8–16 fold as compared to unmodified anoplin).^49^ In addition, anoplin stapled between the second and sixth amino acid is neither hemolytic nor cytotoxic.^49^

Several strategies to develop new antimicrobial agents were based on conjugation of two compounds in order to increase their uptake and activity.^52^ Also, peptide motifs conjugated with antibiotics destabilize bacterial membranes, thereby improving the activity of the components.^53^ Many studies showed that coupling of AMG with amino acids, peptides, peptide nucleic acids, and lipids increased their antimicrobial activity.^18,54,55^ A recent example involves Pentobra, a peptide conjugated with tobramycin, which was found to destabilize the bacterial cell membrane better than tobramycin alone, suggesting that the Pentobra conjugate is more selective against the membrane of *Escherichia coli*.^53^ Another conjugate of NEO with hydrophobic polycarbamates showed a remarkable 256-fold antibacterial activity enhancement against *S. aureus* MRSA ATCC 33592 as compared to unmodified NEO.^56^

Therefore, we hypothesized that conjugating AMG with AMP would enhance the antibacterial activity of AMG, especially against the AMG-resistant strains. We selected NEO and AMK, from the class of 2-deoxystreptamine AMG, and the amphipathic and α-helical anoplin analogues. To determine the effect of the linker, we conjugated the segments through a non-cleavable triazole ring or a cleavable disulfide bond that is cleaved in the presence of a reducing agent, glutathione, found in the bacterial cytoplasm.^57^ For conjugation, we used either the copper-catalyzed alkyne–azide cycloaddition (CuAAC), a click chemistry technique broadly used in bioconjugation of molecules or disulfide bridge formation.^58,59^

In this work, we compare different strategies applied to improve the antibacterial activity including conjugation of AMG antibiotics with AMP, peptide stapling, and the combination of both. We tested the antibacterial and bactericidal activity of the conjugates against different Gram-negative *E. coli* and Gram-positive *S. aureus* strains, including the antibiotic-resistant ones. We also examined the hemolytic activity of the hybrids on sheep red blood cells (RBCs). To the best of our knowledge, this is the first work involving anoplin and its stapled analogue as components of conjugates with antibiotics.

## Results and Discussion

### Design and Synthesis of the AMG and Peptide Conjugates

We proposed two approaches to conjugate AMG and peptides (Figure 1). One was based on the alkyne derivatives of peptides and azide derivatives of AMG and their conjugation using the click chemistry reaction. The other was based on the thiol peptide derivatives and pyridine disulfide NEO and their conjugation via disulfide bond formation. These different conjugation strategies gave either a non-cleavable or cleavable linker between the AMG and peptides. Thus, we could determine whether the stability of the linker in the intracellular environment impacts the antimicrobial activity of these conjugates.

**Scheme 1:**
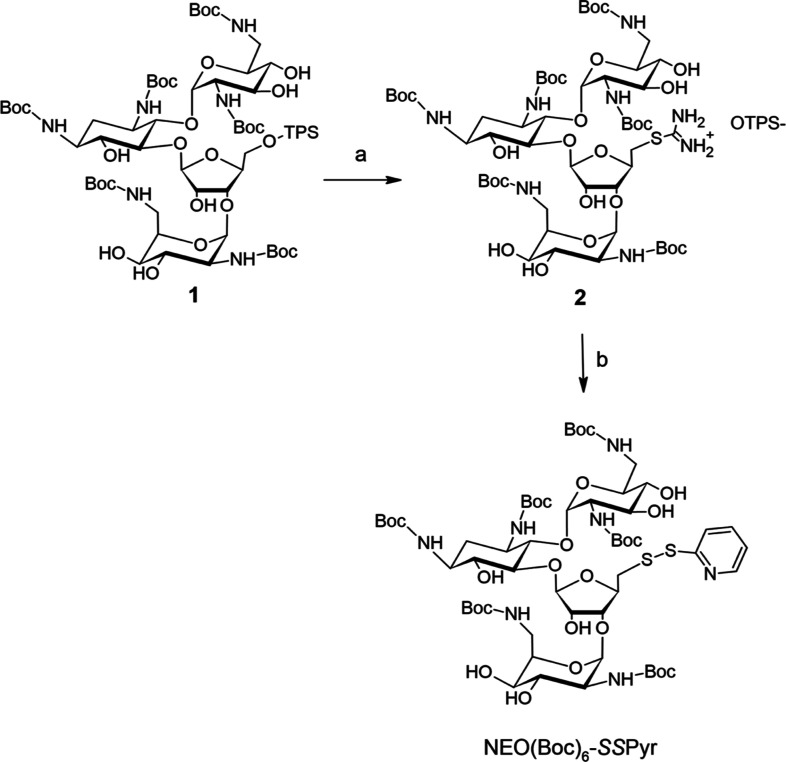
Two-Step Synthesis of the Protected NEO-Pyridyl Disulfide Reagents and conditions: (a) thiourea, EtOH, reflux, 3 days and (b) 2-mercaptopyridine, MeNH_2_, MeOH, rt, 18 h. OTPS^–^—2,4,6 triisopropylbenzenesulfonate.

We used AMK as a representative of the 4,6-disubstituted-2-deoxystreptamines and neomycin B as a representative of the 4,5-disubstituted-2-deoxystreptamines (Figure 1). Both have been investigated for structural modifications.^24,26,27,61^ Neomycin B was selected because of its low price and low biological activity toward several MDR bacteria including the *S. aureus* ATCC BAA1720 MRSA strain.^24^ AMK was chosen because of high resistance of the *E. coli* WR 3551/98 strain to this antibiotic and its commercial availability.^61^ The primary hydroxyl group in these AMGs (C5′ in neomycin B and C6′ in AMK, Figure 1) is preferred for modifications due to its high reactivity and ease of introducing other functional groups.^24,26,27^ Derivatives of AMK and neomycin B were obtained by introducing two different active groups: azide and thiopyridine. The azide derivatives of neomycin B [NEO(Boc)_6_-N_3_] and AMK [AMK(Boc)_4_-N_3_] were synthesized according to the adapted protocols.^27,62,63^ Briefly, all AMG amine groups were protected by a *tert*-butyloxycarbonyl (Boc) group, and then, in a substitution reaction of the triisopropylbenzenesulfonyl group with an azide group, the derivatives were prepared. NEO-pyridyl [NEO(Boc)_6_-SSPyr] was prepared by replacing the triisopropylbenzenesulfonyl group in a two-step reaction to introduce the active thiopyridine group (Scheme 1, details are given in Materials and Methods, Figures S1–S3).

As a peptide for conjugation, we selected anoplin and its hydrocarbon stapled form; both are amidated at the C-terminus for biostability and bear the same net charge of +4e (Figures 1, S4 and S5). We modified the N-terminus of anoplin and anoplin[2-6] by coupling the 10-undecynoic acid and cysteine with the methoxytrityl (Mmt) protecting group. The Mmt group was chosen because of its simple deprotection conditions and orthogonal character with respect to other protecting groups in the peptide sequence.^64^ Appropriately, to conjugate with AMG, alkyne-anoplin and alkyne-anoplin[2-6] were used for the click reaction, while peptides with the additional Cys in the sequence were used to obtain hybrids linked via a disulfide bond (Figures 1, S6 and S7). As a result of the reaction of NEO(Boc)_6_-N_3_ or AMK(Boc)_4_-N_3_ with alkyne-anoplin or alkyne-anoplin[2-6], the 1,2,3-triazole ring was formed (Scheme S1, Table S1, Figures S8–S11).^65^ The triazole ring is not susceptible to hydrolysis, reduction, and oxidation, so it is not cleaved in the bacterial cell, contrary to the disulfide bond.

The second conjugation strategy uses the redox-sensitive formation of disulfide bonds. To avoid the limitations in disulfide bond formation, including side reactions and lower yield due to the appearance of dimeric products, we used the method of forming the disulfide bond on the resin.^58,66,67^ Accordingly, the resins with attached Cys(Mmt)-anoplin or Cys(Mmt)-anoplin[2-6] were treated with a low concentration of trifluoroacetic acid (TFA) solution to remove the Mmt group and finally to obtain the free thiol group at the N-terminus of anoplin and anoplin[2-6].^68^ The NEO-SS-anoplin and NEO-SS-anoplin[2-6] (Scheme S2, Table S1, Figures S12 and S13) were obtained by nucleophilic substitution between the thiol group and active thiopyridine group.^69^

Since there is evidence that peptide dimers can structurally stabilize the natural or engineered peptides and often improve their biological activity,^70^ we also obtained the peptide homo-dimers by the formation of disulfide bonds through the free thiol group at the peptide’s N-terminus (Figures 1, S14, and S15). A disulfide bond was spontaneously formed during the reaction in the aqueous solution.

### Antibacterial Activity

The antibacterial activity of the conjugates was assessed by determining their minimum inhibitory concentration (MIC) and minimum bactericidal concentration (MBC) against Gram-negative *E. coli* (K12 MG 1655 and WR 3551/98) and Gram-positive *S. aureus* (ATCC 29213 and ATCC BAA1720 MRSA) strains (Table 1, Figures S16–S23).

**Table 1:** MIC and MBC of the Conjugates and Their Monomeric Forms on Representative *E. coli* and *S. aureus* Bacterial Strains

	MIC/MBC [μM]							
	*E. coli* K12 MG1655		*E. coli* WR 3551/98		*S. aureus* ATCC 29213		*S. aureus* ATCC BAA1720 MRSA	
conjugates	MIC	MBC	MIC	MBC	MIC	MBC	MIC	MBC
NEO-anoplin	8	8–16	16	16	16	16–32	32	32
NEO-anoplin[2-6]	8	8	16	16	8	16	16	16
AMK-anoplin	16	16	16	16–32	32	>32	>32	>32
AMK-anoplin[2-6]	4	8	16	16	16	16–32	32	≥32
NEO-SS-anoplin	8	8	8	16	4	8	>32	>32
NEO-SS-anoplin[2-6]	8	8–16	8	16	4	8	32	32
amikacin (AMK)	4	8–16	>32	>32	8	8	32	≥32
neomycin (NEO)	4	4	4	8	1	2	>32	>32
anoplin-SS-anoplin	4	8	1	2	16	32	16	32
anoplin[2-6]-SS-anoplin[2-6]	4	4	4	4	16	16	16	16–32
anoplin	32	≥32	>32	>32	>32	>32	>32	>32
anoplin[2-6]	4	8	4	4–8	16	16–32	16	32
NEO + anoplin^a^	4							
NEO + anoplin[2-6]^a^	4				2			
AMK + anoplin^a^	4							
AMK + anoplin[2-6]^a^	4				8			

a MIC values for 1:1 molar mixtures of compounds were derived from checkerboard experiments. For some instances, MIC determination was not possible due to its values exceeding the tested range for at least one of the tested compounds from the mix.

For AMK-anoplin and AMK-anoplin[2-6], we observed at least two-fold MIC decrease (16 μM) on the AMK-resistant *E. coli* WR 3551/98 as compared to AMK alone (MIC > 32 μM). In turn, NEO-anoplin[2-6] and NEO-anoplin inhibited the growth of NEO-resistant *S. aureus* MRSA at concentrations of 16 and 32 μM, respectively. Thus, attachment of unmodified anoplin to AMG makes the antibiotic active against AMG-resistant strains even though anoplin alone is not active against these strains.

Overall, anoplin alone did not inhibit the growth of the selected strains in the tested concentration range up to 32 μM. However, conjugating anoplin to AMK or NEO, in many instances, resulted in a measurable MIC in contrast to free anoplin.

Conjugation of the stapled anoplin[2-6] to AMK or NEO (as in NEO-anoplin[2-6] and AMK-anoplin[2-6]) showed similar or slightly better antibacterial activity, especially against *S. aureus* 29213 and *E. coli* K12, as compared to the conjugates with unstapled anoplin. Anoplin[2-6] proved a helical peptide that effectively penetrated the membrane and cell wall mimics of the *E. coli* K12 strain and showed 4–16 μM MIC against Gram-negative strains.^49^ Therefore, we suppose that the better activity of AMG-anoplin[2-6] conjugates is related to stronger affinity of the stapled peptide to the bacterial cell membrane. Anoplin[2-6] as a permeabilizing agent affects bacterial cell membrane integrity.^49^ We suspect that this disruption of integrity of bacterial membranes by introducing the staple may also facilitate the transport of AMG into the bacteria.

The disulfide bond is often used as a linker between the conjugated segments in hybrid compounds.^57^ Furthermore, the degradability of such linkers in the intra-bacterial environment is used to promote the drug to reach its target.^58,59,71^ Interestingly, NEO conjugates with peptides linked through disulfide bonds (NEO-SS-anoplin and NEO-SS-anoplin[2-6]) inhibited bacterial growth at lower concentrations than the corresponding conjugates with a non-cleavable linker. For the *E. coli* WR 3551/98 strain, it was a two-fold increase in activity. In general, we obtained better antibacterial activities for the conjugates linked through a disulfide bond compared to the same conjugates linked through a non-cleavable triazole ring.

The peptide dimers also showed similar or higher antibacterial activity compared to their monomeric forms. Interestingly, we found a remarkable 32-fold MIC enhancement against *E. coli* WR 3551/98 for the anoplin-SS-anoplin dimer (compared to anoplin monomer), while dimerization of stapled anoplin[2-6] via a disulfide bond showed a similar effect as anoplin[2-6] alone.

In contrast to anoplin[2-6], the dimeric form of anoplin greatly increased antimicrobial activity compared to the monomeric peptide (Table 1). Indeed, it was previously found that the C–C and C–N terminal dimerization of anoplin via a triazole ring, formed in a reaction with additional amino acids introduced at the termini, disrupted the integrity of the bacterial membrane.^45,72^ The flow cytometry experiments proved that these C–C and C–N terminal dimers of anoplin could damage the bacterial membrane.^45,72^ In our work, the increased activity of anoplin dimers is most probably related to the formation of pores in the bacterial lipid membrane.^45,73^ The larger net positive charge (+8e) of dimeric anoplin as compared to the monomeric form increases the binding affinity to the negatively charged bacterial membrane. The anoplin monomer is not helical but can easily adopt the α-helix near the lipids.^48^ In contrast, the stapled anoplin[2-6], whose monomer is already stabilized in a helical form in the buffer solution, in a dimeric form is also helical and thus less structurally flexible, so the improvement in MIC between the stapled monomer and dimer is not so pronounced. Thus, the MIC for the anoplin[2-6] dimer is the same as for its monomeric form.

To make sure the observed effects do not arise solely from the synergy between the unlinked fragments, we investigated the combinatorial effect of non-conjugated NEO and AMK with anoplin and anoplin[2-6] on *E. coli* K12 MG1655 and *S. aureus* ATCC 29213 bacteria strains (Table 2, Figures S24–S26).

**Table 2:** Median FIC Determined from the Individual FIC Indexes within the Entire Checkerboard, as Well as Minimal and Maximal Values of Individual FIC Indexes

	FIC index		
combination	median	minimal value	maximal value
*E. coli* K12 MG1655			
NEO/anoplin	0.75	0.50	2.06
NEO/anoplin[2-6]	1.34	1.13	2.50
AMK/anoplin	1.06	0.75	1.13
AMK/anoplin[2-6]	1.10	1.00	1.25
*S. aureus* ATCC 29213			
NEO/anoplin[2-6]	0.75	0.53	1.13
AMK/anoplin[2-6]	1.19	0.75	2.10

The growth inhibition concentration threshold for anoplin, especially against the *S. aureus* ATCC 29213 strain, was higher than 128 μM, so we did not determine the MIC as it would be irrelevant to pursue larger concentrations. Nevertheless, in the range up to 128 μM, anoplin did not show any synergistic actions with NEO or AMK on *S. aureus*. However, the median fractional inhibitory concentration (FIC), determined from the individual FIC indexes within the entire checkerboard, in the range of 0.5–4 confirmed indifference between anoplin[2-6] and AMK or NEO against both strains. Also, an indifferent effect was observed for the combination of anoplin with NEO or AMK against *E. coli* (Table 2). The minimal value of the FIC index of 0.5 appears only at one particular concentration and does not indicate synergy in the context of the whole checkerboard analysis (Figure S24). Overall, we did not observe any synergy between the molecules used for conjugation.

Data from MIC/MBC and synergy experiments (Tables 1 and 2) revealed that improved inhibitory activity of the conjugates of anoplin variants with antibiotics was a result of the antibiotic activity itself. What is more, if conjugated, these antibiotics usually showed higher MICs than antibiotics mixed with a given anoplin variant.

### Hemolytic Activity

**Figure 2:**
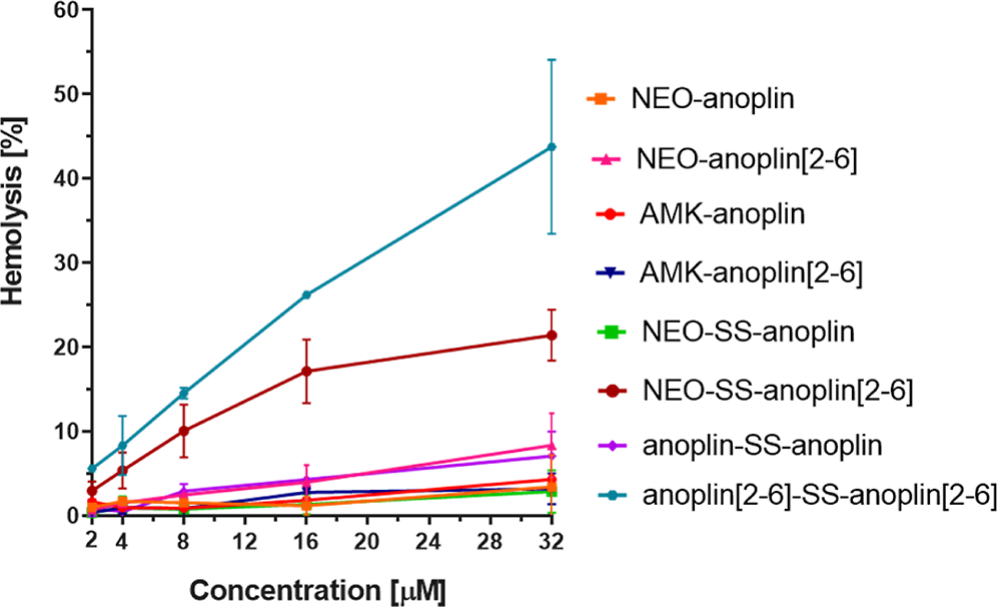
Hemolytic activity of the conjugates against the sheep RBCs. Erythrocytes treated with 1% Triton-X-100 were used as a positive control (100% of hemolysis).

The hemolytic activity of the conjugates against the sheep RBCs is shown in Figure 2. For all conjugates with AMG (except NEO-SS-anoplin[2-6]), the hemolytic activity is negligible at the concentrations of the conjugates that inhibit bacterial growth.

However, conjugates of NEO and anoplin[2-6] show increased hemolytic activity as compared to NEO conjugates with anoplin, while the corresponding conjugates with AMK display lower hemolysis than those with NEO. NEO and AMK amino groups are protonated at pH 7 with the net charge of +6 and +4e, respectively.^11,74−76^ Therefore, the higher hemolytic activity of the NEO-involving compounds could be related to the AMG charge. Still, at the MIC concentrations of these NEO-including conjugates, the hemolysis is not significant.

The anoplin[2-6]-SS-anoplin[2-6] dimer induced most changes in cell viability, with hemolytic activity above 40% at 32 μM, but it was dose-dependent, and at 4 μM (MIC of this dimer for *E. coli*, Table 1), the hemolysis was much lower, slightly exceeding 8%. We have previously found that monomeric anoplin[2-6] at 32 μM displayed only up to 4% hemolysis, so dimerization seems to increase the hemolytic activity for this stapled peptide. However, in general, stapled peptides can be more hemolytic than their unstapled counter-parts due to possibly different charge, hydrophobicity, and stabilized secondary structure.^40,49^

## Conclusions

We designed and synthesized a series of AMG and anoplin conjugates connected via a non-cleavable triazole linker and a cleavable disulfide bond linker. Overall, the conjugates exhibited only slightly enhanced or similar antimicrobial activity as compared to their constituents and overall low hemolytic activity. The NEO conjugates with the cleavable S–S linker (as compared to the non-cleavable linker) have only a two-fold lower MIC (so slightly increased antibacterial activity), suggesting that the peptide may interfere with the binding of AMG to the bacterial ribosome. However, at the same time, regardless of the linker, the peptide contributes to the destabilization of the bacterial cell membrane. We have previously shown that the amphipathicity and helicity of anoplin[2-6] play a crucial role in destabilizing the cell membrane, which may provide an additional entry route for the AMG.^49^ This explains the observed better activity of the conjugates linked through the intracellularly degraded disulfide bond.

Our results also indicate that dimerization of peptides could be potentially beneficial without compromising toxicity. Especially, for the *E. coli* strains, the anoplin-SS-anoplin dimer was at least eight-fold more active than anoplin itself. This may be due to more favorable interactions with the cell wall and ease of membrane permeabilization for the dimeric form. Dimerization of the stapled anoplin did not decrease the MIC and increased hemolytic activity as compared to anoplin[2-6] alone, suggesting that either stapling or dimerization is sufficient to improve the membrane permeability by this peptide.

Our studies suggest that the conjugation of NEO and AMK with anoplin or anoplin[2-6] only modestly improves their activity against AMG-resistant strains. There is no synergistic effect of conjugation over simple mixing of peptide with antibiotic. In general, the stapling strategy shows by far the best improvement of antibacterial activity.

## Materials and Methods

### Materials

All reagents, silica gel, and silica gel plates were purchased from Merck. All chemicals were of analytical or reagent grade, and all buffers were prepared using distilled water. Reactions were monitored by thin-layer chromatography, using silica gel plates (Kieselgel 60F_254_). Column chromatography was performed using silica gel 60 M (0.040–0.063 mm). ESI mass spectra were recorded on an LTQ Orbitrap Velos instrument (Thermo Scientific, Waltham, MA, USA). The synthesized conjugates and peptide derivatives were purified by reverse-phase high-performance liquid chromatography (RP-HPLC) on the analytical column (Knauer C18 columns, 5 μm particles, 4.6 × 250 mm) in different phases gradients (see Table S1) at a flow rate of 1.5 mL/min and wavelength of 220 nm. The mobile phase was composed of 0.1% TFA in acetonitrile (buffer A) and 0.1% TFA in water (buffer B). The presence and purity (>95%) of the obtained compounds were confirmed using RP-HPLC, mass spectrometry, and high-resolution mass spectrometry (Table S1 and Figures S4–S15). The purified products were finally dissolved in 0.1 M HCl, frozen, and lyophilized.

### Peptide Synthesis

Anoplin and anoplin[2-6] were synthesized according to the procedures described by Wojciechowska et al.^49^ Alkyne-anoplin, alkyne-anoplin[2-6], Cys-anoplin, Cys-anoplin[2-6] were obtained by coupling of 10-undecynoic acid or Fmoc-Cys(Mmt)-OH at the N-terminus of anoplin and anoplin[2-6]. These residues, as well as all preceding amino acids, were manually added during solid phase peptide synthesis using 9-fluorenylmethoxycarbonyl (Fmoc) strategies. Cleavage from the resin and purification of the alkyne-anoplin and alkyne-anoplin[2-6] were carried out following the previous protocols.^49^ Cys-anoplin and Cys-anoplin[2-6] were conjugated on the resin (see below).

### Synthesis of Protected AMG-Azide Derivatives

The azide-modified, Boc-protected NEO derivative NEO(Boc)_6_-N_3_ and the azide-modified, Boc-protected AMK derivative AMK(Boc)_4_-N_3_ were synthesized according to the procedure described in refs (27)(62), and (63).

### Protected NEO-Pyridyl Disulfide Synthesis

The pyridine-substituted, disulfide NEO derivative NEO(Boc)_6_-SSPyr was synthesized based on the procedure reported by Wierzba et al.^58^ (Scheme 1). 1,3,2′,6′,2‴,6‴-Hexa-*N*-(*tert*-butoxycarbonyl)-5″-*O*-(2,4,6-triisopropylbenzenesulfonyl)-neomycin (**1**)^62^ (500 mg, 0.36 mmol, 1 equiv) was dissolved in 30 mL of ethanol, thiourea (86 mg, 1.12 mg, 3.1 equiv) was added, and the reaction mixture was refluxed overnight. The reaction progress was monitored by mass spectrometry (*M* = 1481.73 g/mol for the substrate and *M* = 1274.45 g/mol for isothiouronium cation). Next day, additional portion of thiourea (86 mg, 1.12 mg, 3.1 equiv) was added, and reflux was continued overnight. Finally, the third portion of thiourea (86 mg, 1.12 mg, 3.1 equiv) was added, and reflux was continued for the third day. When the substrate mass peak was no longer observed in the mass spectrum, the solvent was evaporated, and the crude product as an isothiouronium salt (**2**) was used in the next step without further purification.

The obtained NEO isothiouronium sulfonate salt (**2**) (0.36 mmol, 100% yield assumed) and 2-mercaptopyridine (81 mg, 0.72 mmol, 2 equiv) were dissolved in 15 mL of MeOH, and 2 M solution of MeNH_2_ (∼2 mL, 3.6 mmol, 10 equiv) was added. The reaction mixture was stirred at room temperature for 18 h, then evaporated with small amount of silica gel under reduced pressure. The final product NEO(Boc)_6_-SSPyr was isolated and purified by column chromatography using 5% solution of MeOH in CHCl_3_ followed by 10% solution of MeOH in CHCl_3_. Yield: 305 mg (63%).

### Synthesis of the AMG-Peptide Conjugates by the CuAAC Reaction

The following conjugates NEO-anoplin, NEO-anoplin[2-6], AMK-anoplin, and AMK-anoplin[2-6] were synthesized using CuAAC. The CuAAC reaction components (azide-AMG/alkyne-peptide/CuSO_4_·5H_2_O/sodium l-ascorbate) were used in a molar ratio (1:2:2:8). The AMK(Boc)_4_-N_3_ or NEO(Boc)_6_-N_3_ (2 μmol) with alkyne-anoplin or alkyne-anoplin[2-6] (4 μmol) was dissolved in a solution of water and *t*-butanol (1 mL) (2:1, v/v). Freshly prepared water solutions of CuSO_4_·5H_2_O (4 μmol, 0.1 M) and sodium ascorbate (32 μmol, 0.5 M) were added. The mixture was stirred at 30 °C in a thermoshaker at 600 rpm for 24 h. After lyophilization, the removal of the Boc-protective groups from AMG derivatives was performed by treatment in a solution of TFA/triisopropylsilane (TIPS)/*m*-cresol (95:2.5:2.5; v/v/v) and mixed for 30 min. After adding cold diethyl ether, the conjugates were precipitated, decanted, then dissolved in water, lyophilized, and subsequently purified by analytical RP-HPLC.

### Synthesis of AMG-Peptide Conjugates by Disulfide Bond Formation

Conjugation of NEO-SSPyr with Cys-anoplin or Cys-anoplin[2-6] by disulfide bond formation was performed on the resin (TentaGel S RAM resin; amine groups loading of 0.24 mmol/g).^66^ After the synthesis of peptides, the Fmoc deprotection from the N-terminus was carried out using 20% piperidine in dimethylformamide (DMF) for two cycles in 10 min. The Mmt protective group on Cys was removed by adding the solution of dichloromethane (DCM)/TFA/TIPS (94:1:5) for five cycles in 2 min.^77^ The resins were washed with DCM and DMF solution under a nitrogen atmosphere. Three-fold molar excess of NEO-SSPyr was dissolved in the DMF/*N*-methylpyrrolidone (NMP); 1:1; v/v and mixed for 3 h. Coupling was repeated with a fresh portion of NEO-SSPyr in a two-fold molar excess and carried out for another 3 h. Removal of the protecting groups and cleavage of the conjugates from the resin were performed by treatment with a TFA/water/TIPS (95:2.5:2.5; v/v/v) mixture for 3 h. Conjugates were precipitated by adding cold diethyl ether, decanted, then dissolved in water, lyophilized, and finally purified by analytical RP-HPLC.

### Synthesis of Peptide Dimers

Cys-anoplin and Cys-anoplin[2-6] were cleaved from the resin by treatment with a TFA/phenol/TIPS/water (95:2:2:1; v/v/v/v) for 3 h. By adding cold diethyl ether, the peptides with the free thiol group were precipitated, decanted, and dissolved in water. The anoplin-SS-anoplin and anoplin[2-6]-SS-anoplin[2-6] peptide dimers were formed in the solution of water under an air atmosphere spontaneously by disulfide bond formation.^78^ Finally, the products were purified by analytical PR-HPLC and lyophilized.

### Antibacterial Activity Determination

The MIC values were determined as follows. Bacteria were first cultured overnight in 2 mL of lysogeny broth (LB, VWR Chemicals) at 37 °C with shaking. Next, 20 μL of overnight culture was transferred into 2 mL of Miller-Hinton broth (MHB, Difco) medium and further cultured at 37 °C with shaking until the culture reached OD_600_ = 0.3. Subsequently, the culture was diluted 1:100 in a fresh MHB medium, and aliquots of 50 μL were mixed with 50 μL of previously prepared dilutions of the tested compound in MHB on a transparent flat-bottom 96-well plate (Nest). The plate was then sealed with transparent foil (Titer-Tops) and incubated for 20 h, at 37 °C with shaking. Following incubation, optical density at 600 nm was measured using a Tecan Sunrise plate reader. Growth inhibition was determined by comparing the given sample with untreated culture (growth control—GC) with MHB alone (sterility control—SC) as an additional reference. The experiment was conducted in at least two biological replicates of two technical replicates each. Statistical significance was determined by the two-way ANOVA test using GraphPad Prism 9 software.

The MBC values were determined by diluting wells from the MIC experiment plate in fresh MHB medium. Dilutions of 10-, 100-, and 1000-times were prepared for the MIC well and up to two-folds higher than MIC concentrations of a given compound. Dilutions were made on a transparent flat-bottom 96-well plate (Nest), which was then sealed and incubated for 24 h, at 37 °C with shaking. The growth of a given sample well was compared with GC and SC controls. A particular concentration of a compound was considered bactericidal if no growth was observed for at least 100- and 1000-times dilutions.

### Evaluation of the Synergistic Effect

The synergy between the compounds was assessed using the checkerboard method in a similar way as mentioned in our previous study.^79,80^ Bacteria were first cultured overnight in 2 mL of LB (VWR Chemicals) at 37 °C with shaking. Next, 20 μL of overnight culture was transferred into 2 mL of MHB medium and further cultured at 37 °C with shaking until the culture reached OD_600_ = 0.3. Subsequently, culture was diluted 1:200 in a fresh MHB medium, and aliquots of 90 μL were mixed with 10 μL of previously prepared dilutions of tested compounds in MHB on a transparent flat-bottom 96-well plate (Nest). The plate was then sealed with transparent adhesive foil (Titer-Tops) and incubated for 20 h, at 37 °C with shaking. Following incubation, optical density at 600 nm was measured using the Tecan Sunrise plate reader. Growth inhibition was determined by comparing the given sample with untreated culture (GC) with MHB alone (SC) as an additional reference. The FIC index was calculated for the first cell within each row and column where growth inhibition was observed. The following formula was used
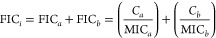
*C*_*a*_, *C*_*b*_—concentration of the agent in combination, and MIC_*a*_, MIC_*b*_—MICs for agent *a* and agent *b* alone.

Then, the median FIC was determined for the entire checkerboard. In addition, the minimal and maximal values were distinguished (Table 2). The FIC values ≤ 0.5 were considered as synergy, 0.5< and ≤4 as indifference, and >4 as antagonism.

### Hemolytic Activity

The hemolytic activity of the peptides against intact erythrocytes was tested using sheep fresh RBCs. The 200 μL of sheep RBCs was washed three times with PBS buffer (10 mM, pH 7.4) at 3500 rpm for 5 min. Then, the cells were diluted in 10 mL of PBS buffer, divided into 200 μL of aliquots in 1.5 mL tubes, and pelleted by centrifugation. The concentration series of AMG–AMP conjugates and peptide dimers was prepared in PBS buffer. 200 μL of each concentration (32, 16, 8, 4, and 2 μM) was added to RBC suspension and incubated for 30 min (165 rpm, 37 °C). Finally, the cells with each incubated sample were centrifuged (3500 rpm, 5 min). Then, 100 μL of the supernatant from each tube was collected into a clear 96-well plate. The sample absorbance was measured at 405 nm using a spectrophotometer (Microplate Reader BioTek, Winooski, VT, United States). The hemolytic activity, as the percentage of hemolysis, was calculated from the following equation
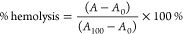
where *A*_0_ is the absorbance intensity of the RBC in buffer (background), *A* is the absorbance intensity of the RBS in the presence of peptides, and *A*_100_ is the absorbance intensity of the Triton X-100.

The erythrocyte suspension treated with 1% Triton X-100 served as a positive control, and the untreated suspension was used as a negative control. Tests were performed with duplicate samples, and the average values of two independent measurements were recorded..

## Supporting Information Available

MS spectra and RP-HPLC chromatograms of AMG-derivatives, peptide, peptide-derivatives, AMG–AMP conjugates, and peptide dimers; synthesis schemes of the AMG–peptide conjugates; optical density (OD_600_) as a measure of *E. coli* K-12 MG1655, *E. coli* WR 3551/98, *S. aureus* ATCC 29213, and *S. aureus* BAA-1720 MRSA growth after 20 h incubation with AMG–AMP conjugates, peptide dimers, amikacin, and neomycin; and typical checkerboard test combinations determined for *E. coli* K12 MG1655 and *S. aureus* ATCC 29213 (PDF)

## Abbreviations

AMG: aminoglycosides
AMK: amikacin
AMP: antimicrobial peptides
Boc: *tert*-butyloxycarbonyl
CuAAC: copper-catalyzed alkyne–azide cycloaddition
*E. coli*: *Escherichia coli*
DMF: dimethylformamide
FIC: fractional inhibitory concentration
Fmoc: 9-fluorenylmethoxycarbonyl
NEO: neomycin
NMP: *N*-methylpyrrolidone
MBC: minimal bactericidal concentration
MIC: minimal inhibitory concentration
Mmt: methoxytrityl
RP-HPLC: reverse-phase high-performance liquid chromatography
*S. aureus*: *Staphylococcus aureus*
TFA: trifluoroacetic acid
TIPS: triisopropylsilane
