# Supplementary material for: Conjugates of Aminoglycosides with Stapled Peptides as a Way to Target Antibiotic-Resistant Bacteria
Source: ACS Omega. 2023 May 16;8(21):19047–56. doi: 10.1021/acsomega.3c02071 (PMC10233823; doi:10.1021/acsomega.3c02071)
Supplement: Supplementary file 1 — ao3c02071_si_001.pdf [file ao3c02071_si_001.pdf]

Supporting Information for:

Conjugates of aminoglycosides with stapled peptides as a way to target antibiotic resistant bacteria

Julia Macyszyn[§], Michał Burmistrz[§], Adam Mieczkowski[±], Monika Wojciechowska[§,*], Joanna Trylska[§,*]

[§]Centre of New Technologies, University of Warsaw, Banacha 2c, 02-097 Warsaw, Poland
[±]Institute of Biochemistry and Biophysics, Polish Academy of Sciences, Pawinskiego 5a, 02-106 Warsaw, Poland

*Correspondence:
Monika Wojciechowska, m.wojciechowska@cent.uw.edu.pl
Joanna Trylska, joanna@cent.uw.edu.pl

Table of Contents

Supplementary Figures

Figure S1. The mass spectrum, chemical structure, calculated mass and RP-HPLC chromatogram of crude fraction of AMK(Boc)$_4$-N$_3$.

Figure S2. The mass spectrum, chemical structure, calculated mass and RP-HPLC chromatogram of crude fraction of NEO(Boc)$_6$-N$_3$.

Figure S3. The mass spectrum, chemical structure, calculated mass and RP-HPLC chromatogram of crude fraction of NEO(Boc)$_6$-*SS*Pyr.

Figure S4. The RP-HPLC chromatogram after purification, calculated mass, chemical structures and high-resolution mass spectrum of anoplin.

Figure S5. The RP-HPLC chromatogram after purification, calculated mass, chemical structures and high-resolution mass spectrum of anoplin[2-6].

Figure S6. The mass spectrum, chemical structures, calculated mass and RP-HPLC chromatogram of alkyne-anoplin after purification.

Figure S7. The mass spectrum, chemical structures, calculated mass and RP-HPLC chromatogram of alkyne-anoplin[2-6] after purification.

Figure S8. The RP-HPLC chromatogram after purification, calculated mass, chemical structures and high-resolution mass spectrum of NEO-anoplin.

Figure S9. The RP-HPLC chromatogram after purification, calculated mass, chemical structures and high-resolution mass spectrum of NEO-anoplin[2-6].

Figure S10. The RP-HPLC chromatogram after purification, calculated mass, chemical structures and high-resolution mass spectrum of AMK-anoplin.

Figure S11. The RP-HPLC chromatogram after purification, calculated mass, chemical structures and high-resolution mass spectrum AMK-anoplin[2-6].

Figure S12. The RP-HPLC chromatogram after purification, calculated mass, chemical structures and high-resolution mass spectrum NEO-*SS*-anoplin.

Figure S13. The RP-HPLC chromatogram after purification, calculated mass, chemical structures and high-resolution mass spectrum NEO-*SS*-anoplin[2-6].

Figure S14. The RP-HPLC chromatogram after purification, calculated mass, chemical structures and high-resolution mass spectrum of anoplin-*SS*-anoplin.

Figure S15. The RP-HPLC chromatogram after purification, calculated mass, chemical structures and high-resolution mass spectrum of anoplin[2-6]-*SS*-anoplin[2-6].

Scheme S1. Synthesis of the AMG-peptide conjugates: a) NEO-anoplin, b) NEO-anoplin[2-6], c) AMK-anoplin and d) AMK-anoplin[2-6] by CuAAC reaction.

Scheme S2. Synthesis of the AMG-peptide conjugates a) NEO-*SS*-anoplin, b) NEO-*SS*-anoplin[2-6] by disulfide bond formation on the resin.

Table S1. Retention times, molecular masses and yields of the synthesized compounds.

Figure S16. Optical density (OD_{600}) as a measure of *E. coli* K-12 MG1655 growth shown after 20 h incubation with various concentrations of: NEO-anoplin, NEO-anoplin[2-6], AMK-anoplin, AMK-anoplin[2-6], anoplin-*SS*-anoplin and anoplin[2-6]-*SS*-anoplin[2-6]. GC – growth control, SC – sterility control. Error bars represent the standard error of the mean; n=3. Statistical significance between the samples and GC: **** P < 0.0001, ** P < 0.01, ns – not significant.

Figure S17. Optical density (OD_{600}) as a measure of *E. coli* K-12 MG1655 growth shown after 20 h incubation with various concentrations of: NEO-*SS*-anoplin, NEO-*SS*-anoplin[2-6], amikacin, neomycin, anoplin and anoplin[2-6]. GC – growth control, SC – sterility control. Error bars represent the standard error of the mean; n=3. Statistical significance between the samples and GC: **** P < 0.0001, *** P < 0.001, ns – not significant.

Figure S18. Optical density (OD_{600}) as a measure of *E. coli* WR 3551/98 growth shown after 20 h incubation with various concentrations of: NEO-anoplin, NEO-anoplin[2-6], AMK-anoplin, AMK-anoplin[2-6], anoplin-*SS*-anoplin and anoplin[2-6]-*SS*-anoplin[2-6]. GC – growth control, SC – sterility control. Error bars represent the standard error of the mean; n=3. Statistical significance between the samples and GC: **** P < 0.0001, *** P < 0.001, ns – not significant.

Figure S19. Optical density (OD_{600}) as a measure of *E. coli* WR3551/98 growth shown after 20 h incubation with various concentrations of: NEO-*SS*-anoplin, NEO-*SS*-anoplin[2-6], amikacin, neomycin, anoplin and anoplin[2-6]. GC – growth control, SC – sterility control. Error bars represent the standard error of the mean; n=3. Statistical significance between the samples and GC: **** P < 0.0001, ** P < 0.01, ns – not significant.

Figure S20. Optical density (OD_{600}) as a measure of *S. aureus* ATCC 29213 growth shown after 20 h incubation with various concentrations of: NEO-anoplin, NEO-anoplin[2-6], AMK-anoplin, AMK-anoplin[2-6], anoplin-*SS*-anoplin and anoplin[2-6]-

SS-anoplin[2-6]. GC – growth control, SC – sterility control. Error bars represent the standard error of the mean; n=3. Statistical significance between the samples and GC: **** P < 0.0001, ** P < 0.01, * P < 0.05, ns – not significant.

Figure S21. Optical density (OD_{600}) as a measure of *S. aureus* ATCC 29213 growth shown after 20 h incubation with various concentrations of: NEO-*SS*-anoplin, NEO-*SS*-anoplin[2-6], amikacin, neomycin, anoplin and anoplin[2-6]. GC – growth control, SC – sterility control. Error bars represent the standard error of the mean; n=3. Statistical significance between the samples and GC: **** P < 0.0001, *** P < 0.001 ** P < 0.01, ns – not significant.

Figure S22. Optical density (OD_{600}) as a measure of *S. aureus* BAA-1720 MRSA growth shown after 20 h incubation with various concentrations of: NEO-anoplin, NEO-anoplin[2-6], AMK-anoplin, AMK-anoplin[2-6], anoplin-*SS*-anoplin and anoplin[2-6]-*SS*-anoplin[2-6]. GC – growth control, SC – sterility control. Error bars represent the standard error of the mean; n=3. Statistical significance between the samples and GC: **** P < 0.0001, ns – not significant.

Figure S23. Optical density (OD_{600}) as a measure of *S. aureus* BAA-1720 MRSA growth shown after 20 h incubation with various concentrations of: NEO-*SS*-anoplin, NEO-*SS*-anoplin[2-6], amikacin, neomycin, anoplin and anoplin[2-6]. GC – growth control, SC – sterility control. Error bars represent the standard error of the mean; n=3. Statistical significance between the samples and GC: **** P < 0.0001, ns – not significant.

Figure S24. Typical checkerboard test combinations of A) NEO and anoplin and B) NEO and anoplin[2-6] determined for *E. coli* K12 MG1655 from one replicate. The calculated FIC index shows no synergistic effect but an additive interaction of the tested combination. White wells indicate no growth and dark grey indicate visible growth. Light grey indicates partial growth.

Figure S25. Typical checkerboard test combinations of A) AMK and anoplin and B) AMK and anoplin[2-6] determined for E. *coli* K12 MG1655 from one replicate. The calculated FIC index shows no synergistic effect but an additive interaction of the tested combination. White wells indicate no growth and dark grey indicate visible growth. Light grey indicates partial growth.

Figure S26. Typical checkerboard test combinations of A) NEO and anoplin[2-6] and B) AMK and anoplin[2-6] determined for *S. aureus* ATCC 29213 from one replicate. The calculated FIC index shows no synergistic effect but an additive interaction of the tested combination. White wells indicate no growth and dark grey indicate visible growth.

Supplementary Figures

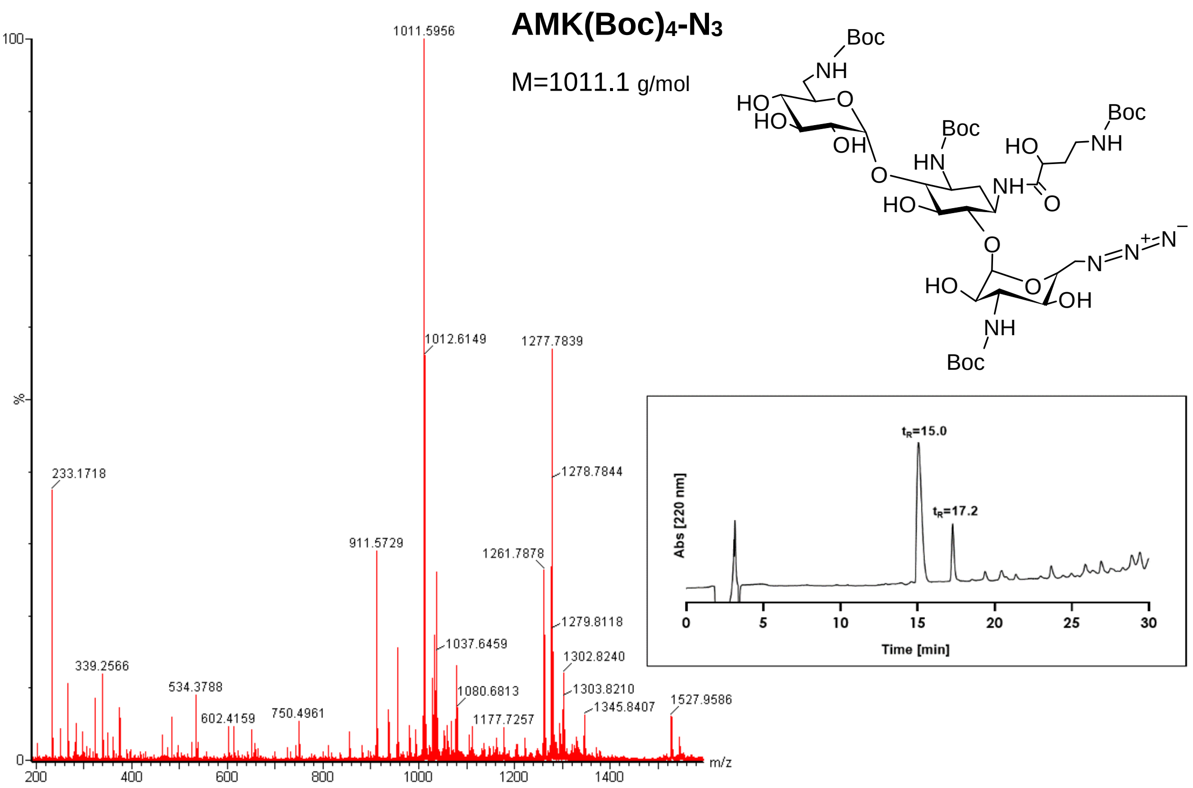

Figure S1. The mass spectrum, chemical structures, calculated mass and RP-HPLC chromatogram of AMK(Boc)$_4$-N$_3$.

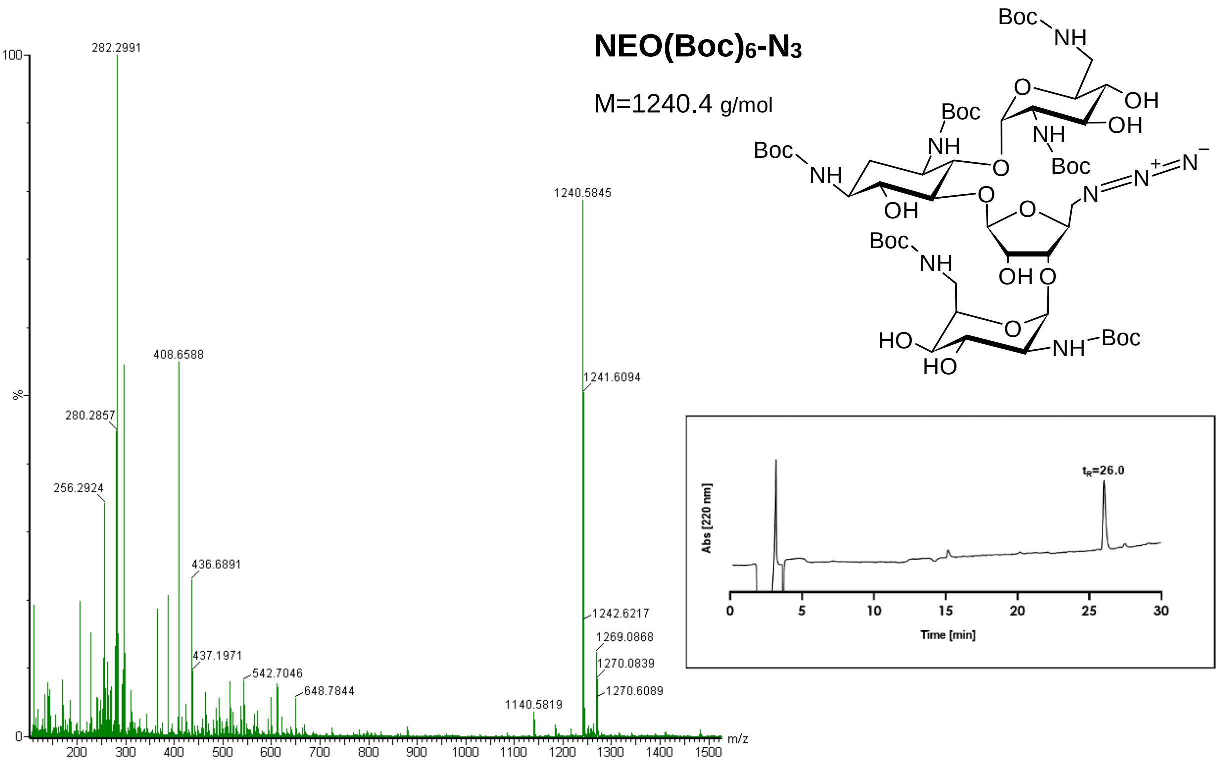

Figure S2. The mass spectrum, chemical structures, calculated mass and RP-HPLC chromatogram of NEO(Boc)$_6$-N$_3$.

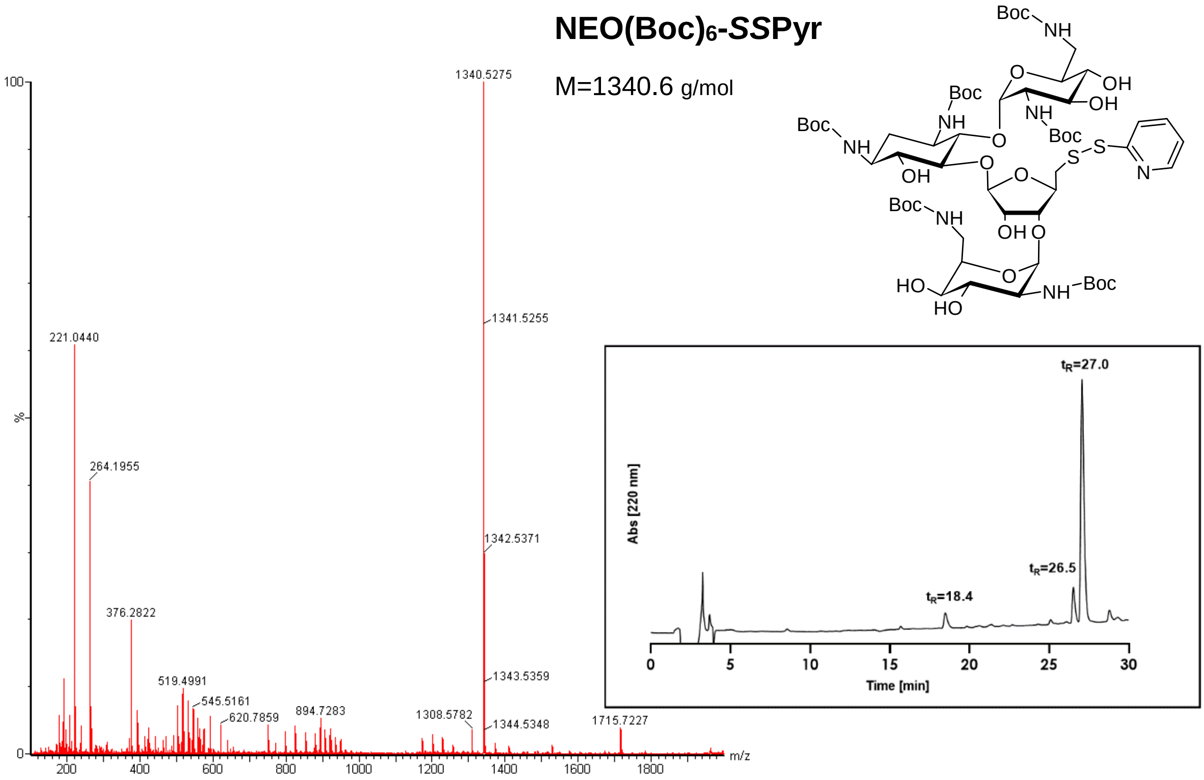

Figure S3. The mass spectrum, chemical structures, calculated mass and RP-HPLC chromatogram of NEO(Boc)$_6$-*SS*Pyr.

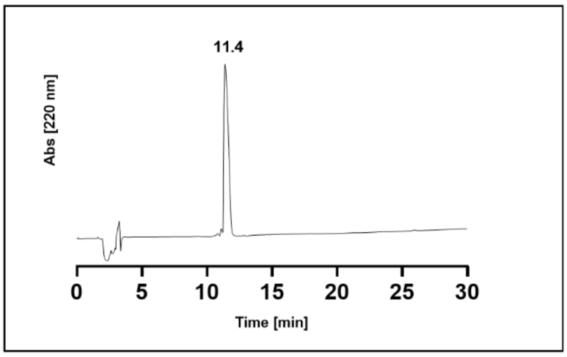

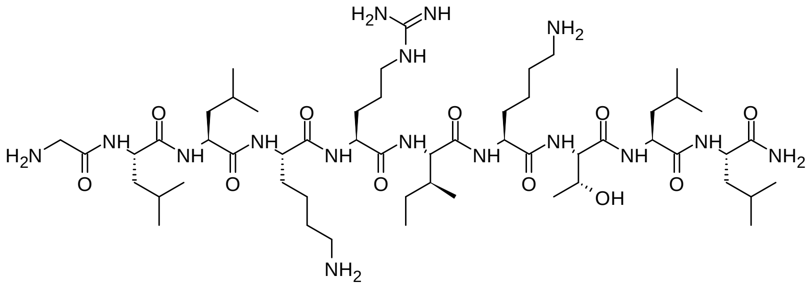

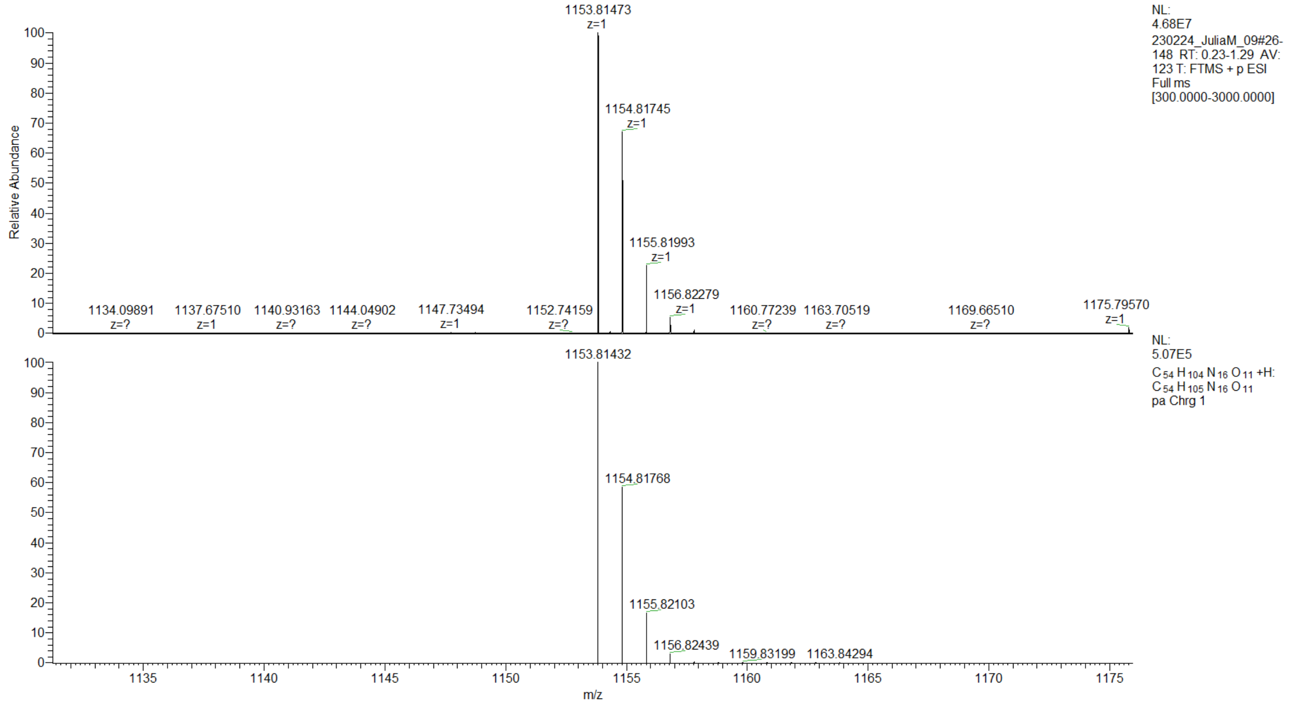

Figure S4. The RP-HPLC chromatogram after purification, calculated mass, chemical structures and high-resolution mass spectrum of anoplin.

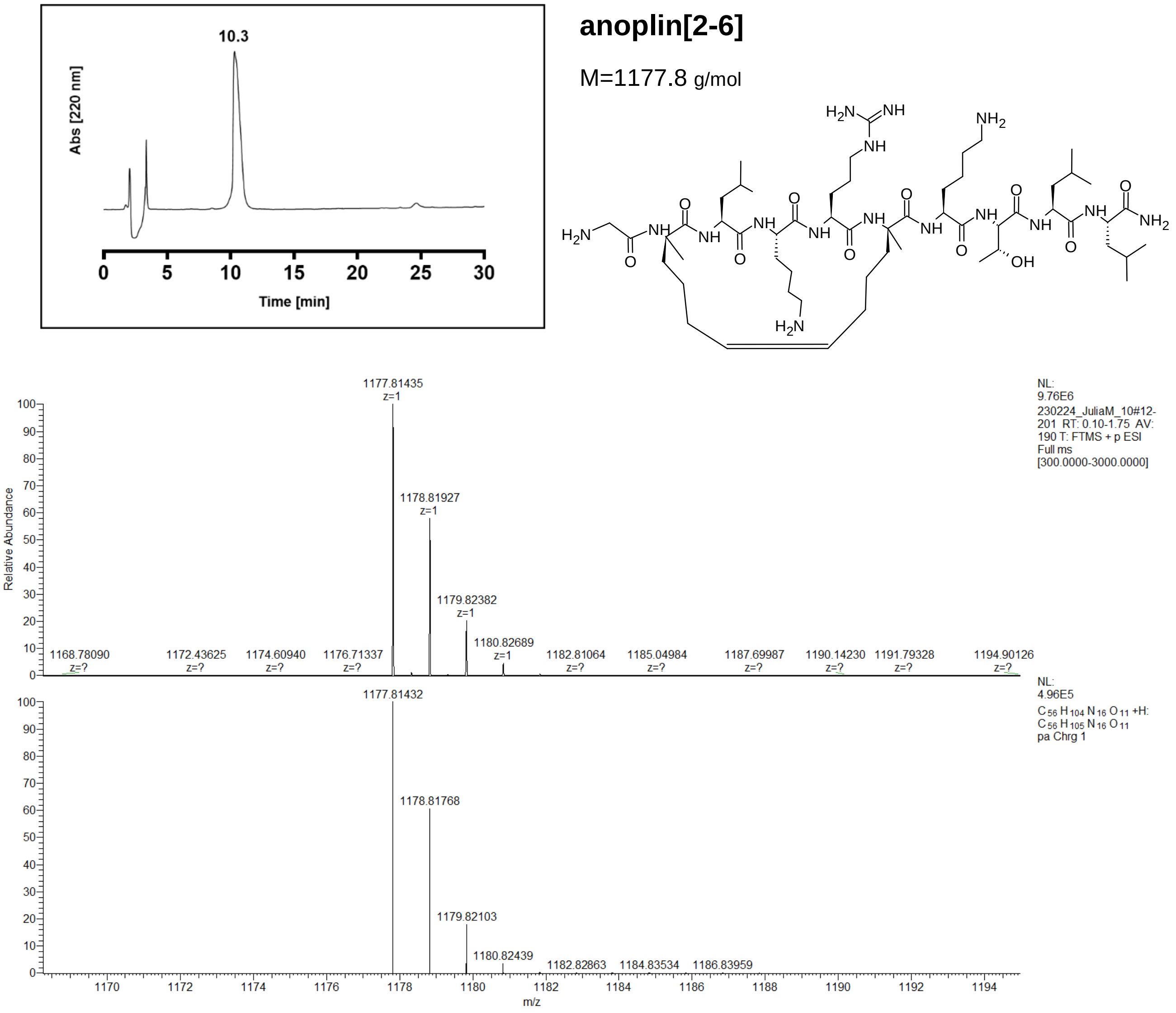

Figure S5. The RP-HPLC chromatogram after purification, calculated mass, chemical structures and high-resolution mass spectrum of anoplin[2-6].

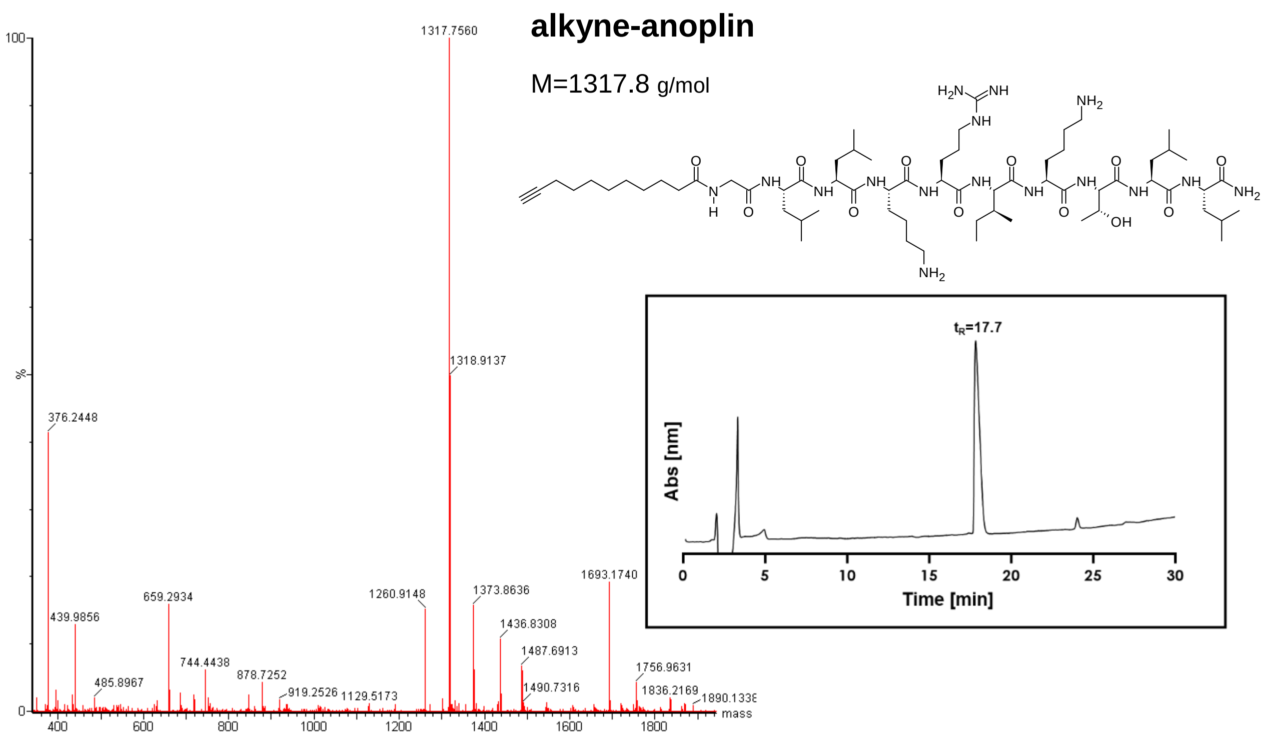

Figure S6. The mass spectrum, chemical structures, calculated mass and RP-HPLC chromatogram of alkyne-anoplin after purification.

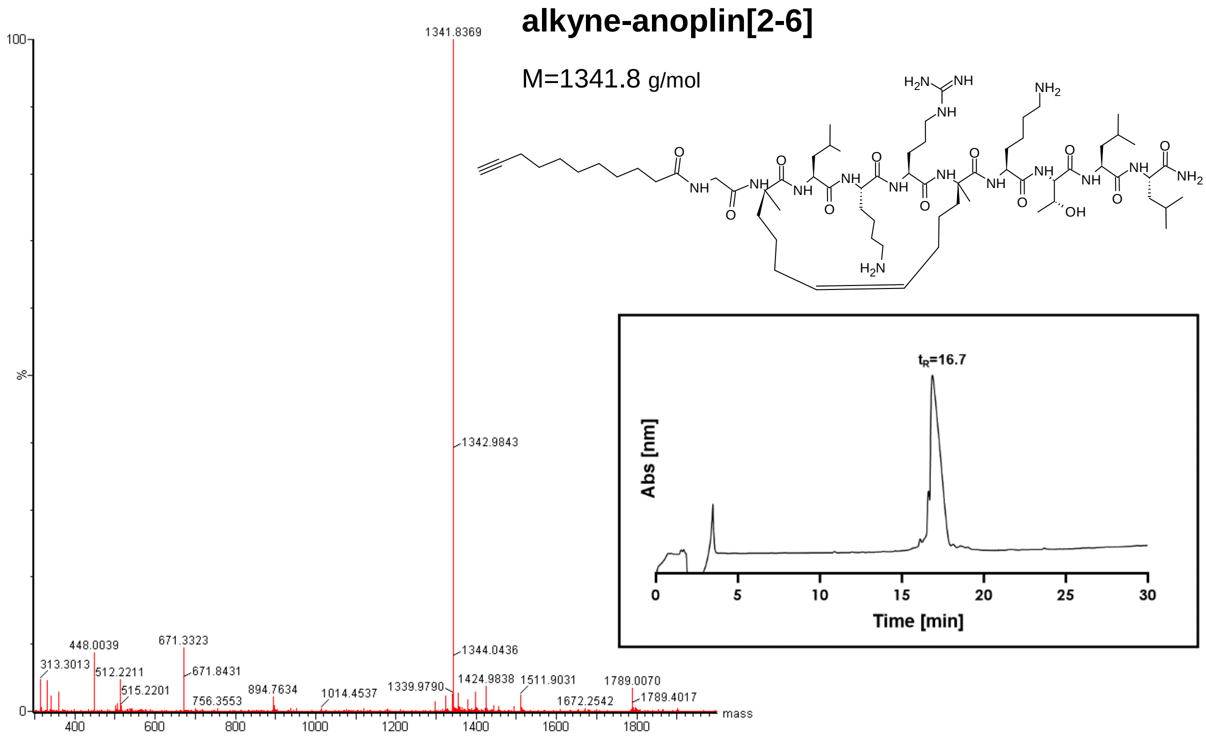

Figure S7. The mass spectrum, chemical structures, calculated mass and RP-HPLC chromatogram of alkyne-anoplin[2-6] after purification.

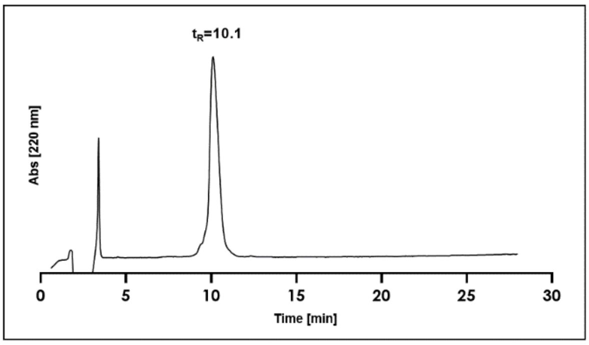

NEO-anoplin

M=1957.3 g/mol

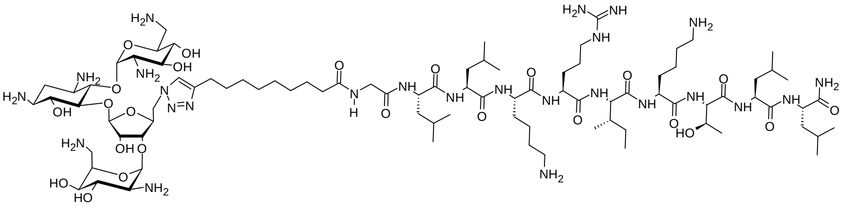

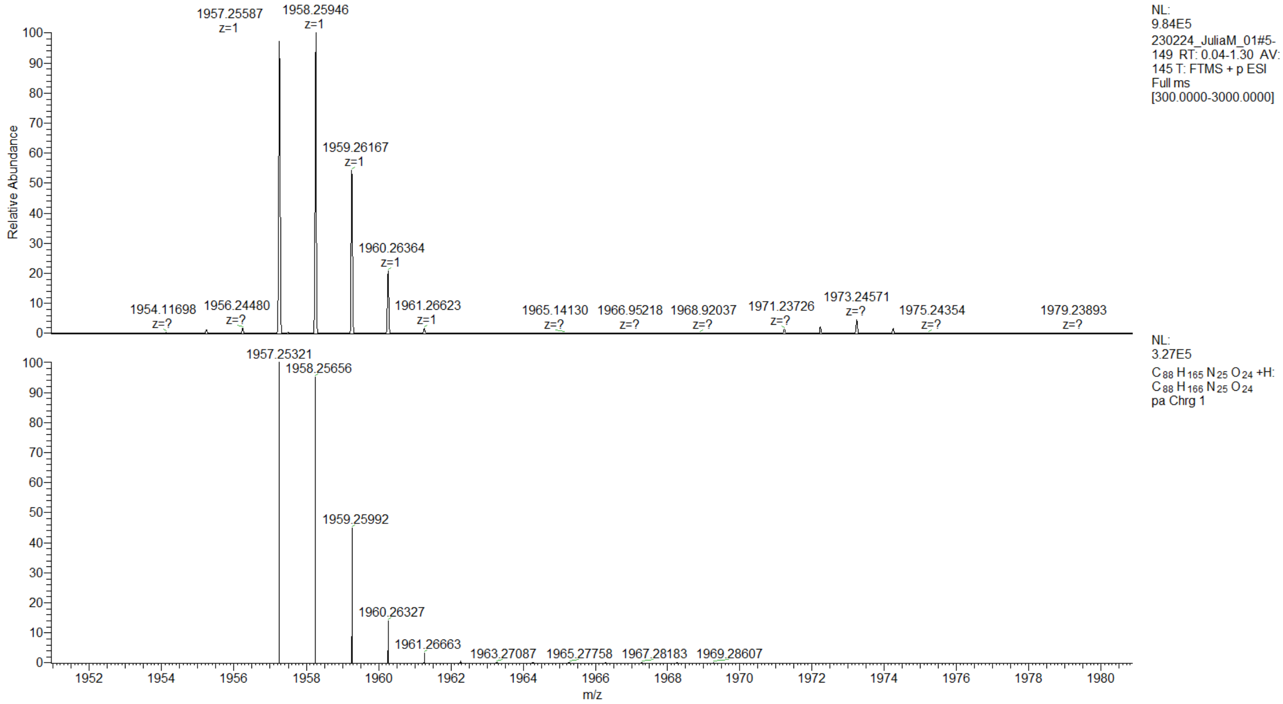

Figure S8. The RP-HPLC chromatogram after purification, calculated mass, chemical structures and high-resolution mass spectrum of NEO-anoplin.

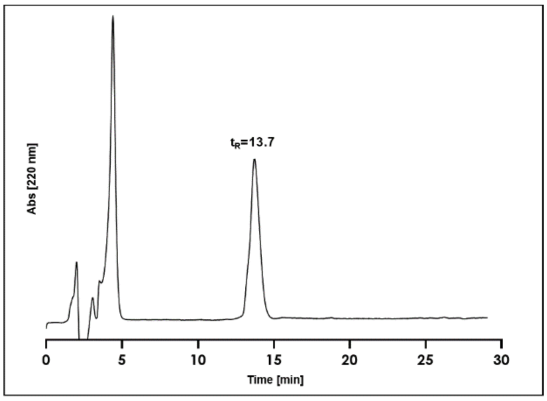

NEO-anoplin[2-6]

M=1981.3 g/mol

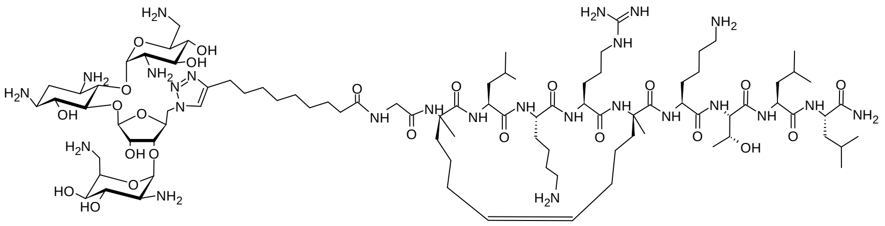

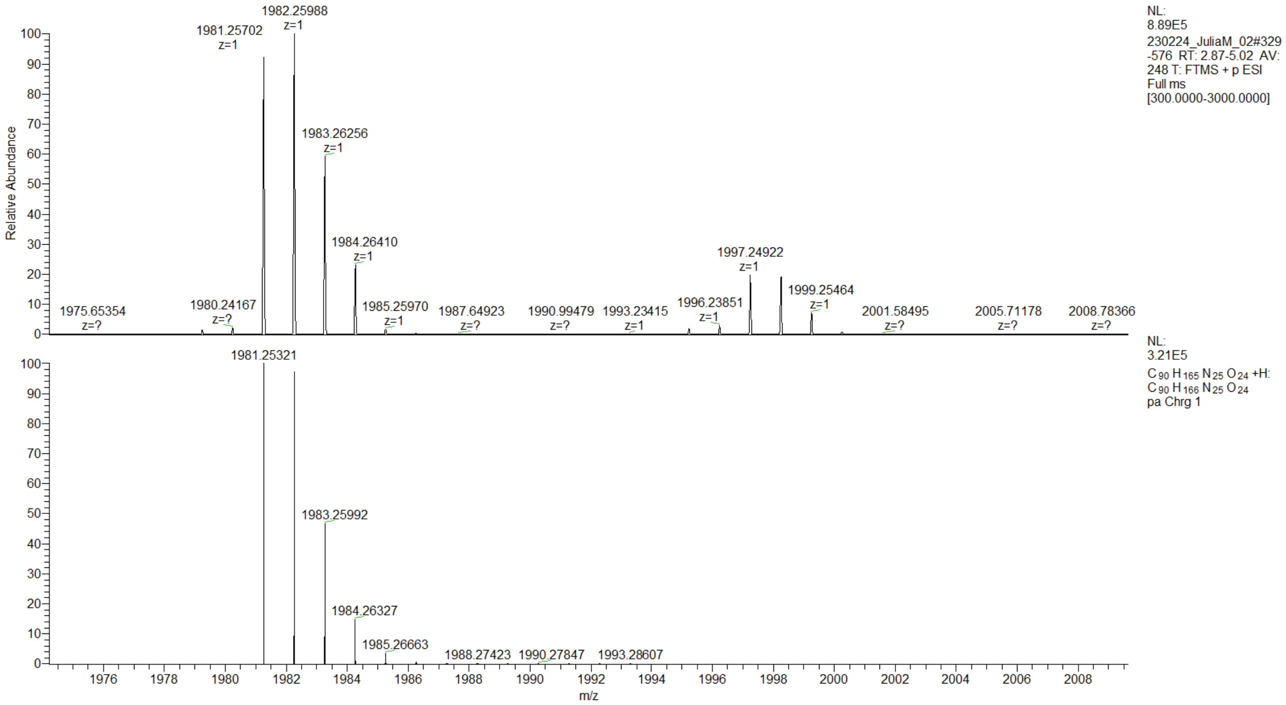

Figure S9. The RP-HPLC chromatogram after purification, calculated mass, chemical structures and high-resolution mass spectrum of NEO-anoplin[2-6].

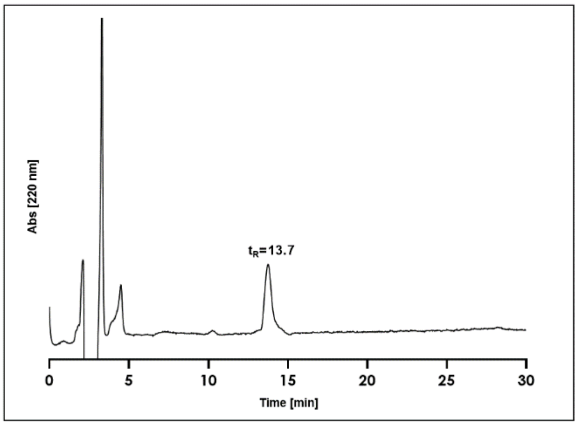

AMK-anoplin

M=1928.2 g/mol

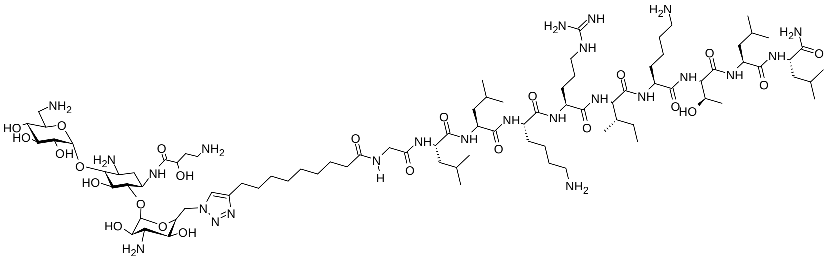

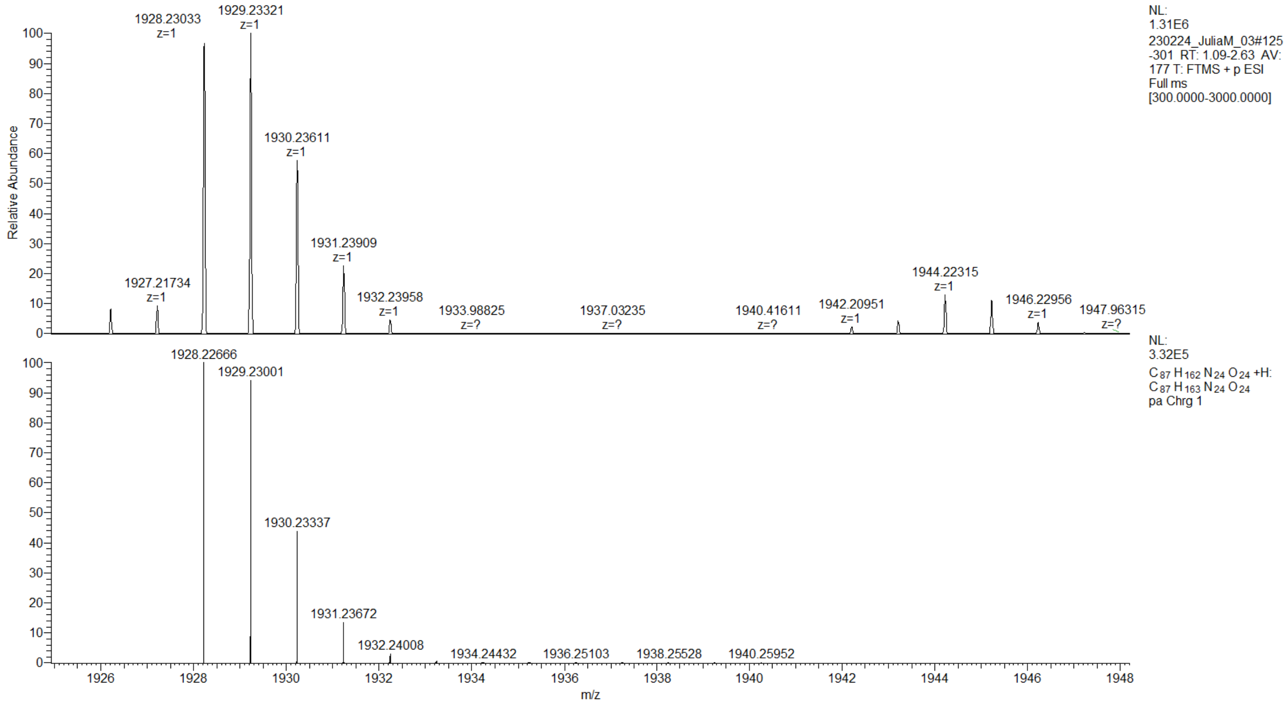

Figure S10. The RP-HPLC chromatogram after purification, calculated mass, chemical structures and high-resolution mass spectrum of AMK-anoplin.

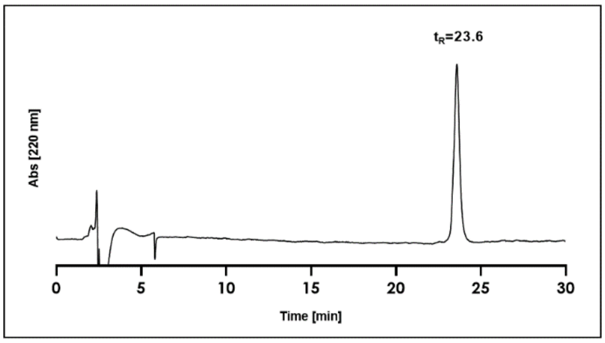

AMK-anoplin[2-6]

M=1952.2 g/mol

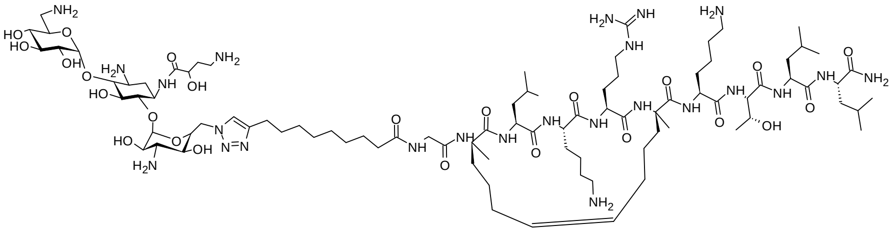

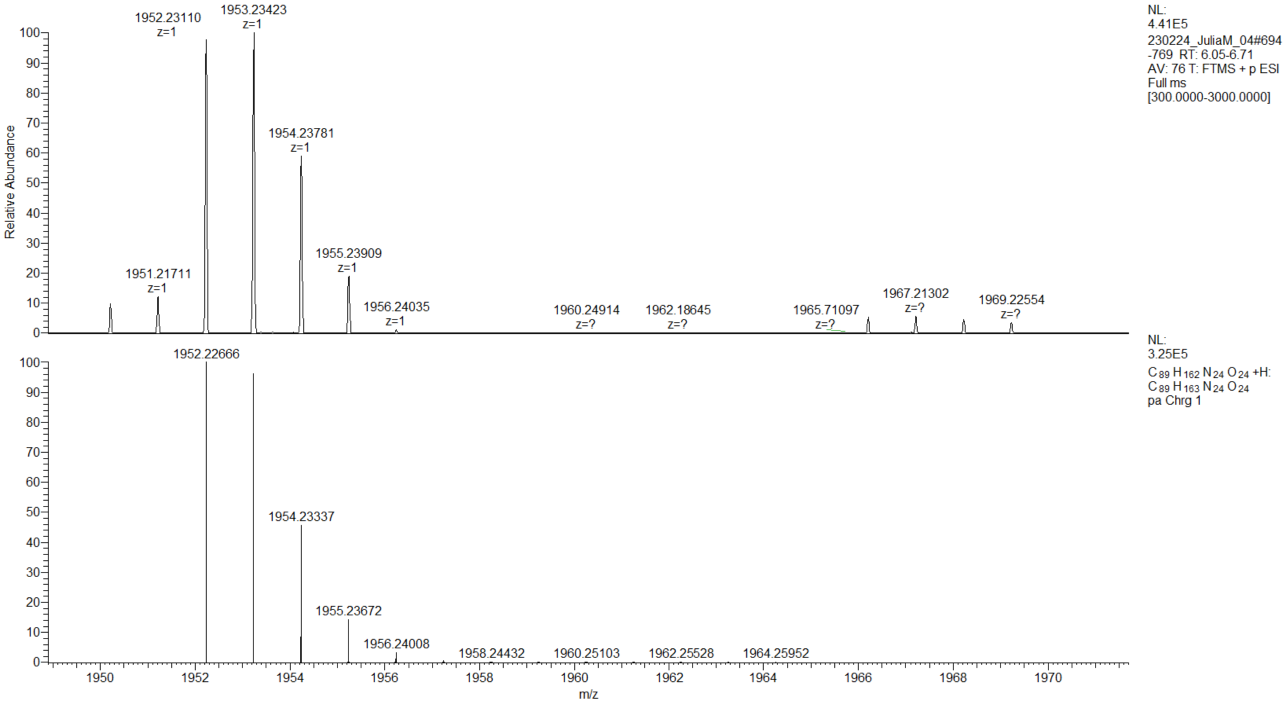

Figure S11. The RP-HPLC chromatogram after purification, calculated mass, chemical structures and high-resolution mass spectrum of AMK-anoplin[2-6].

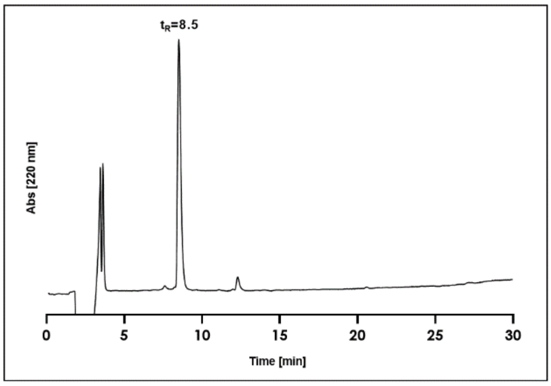

NEO-*SS*-anoplin

M=1885.1 g/mol

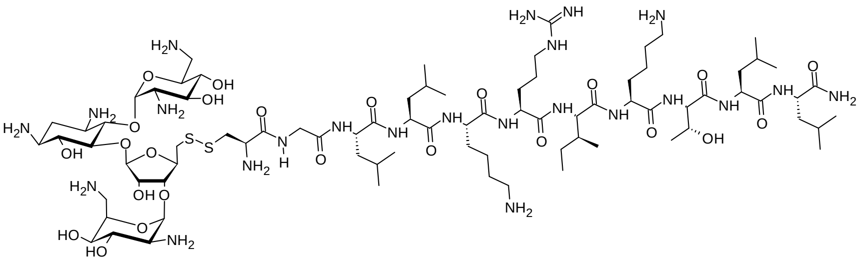

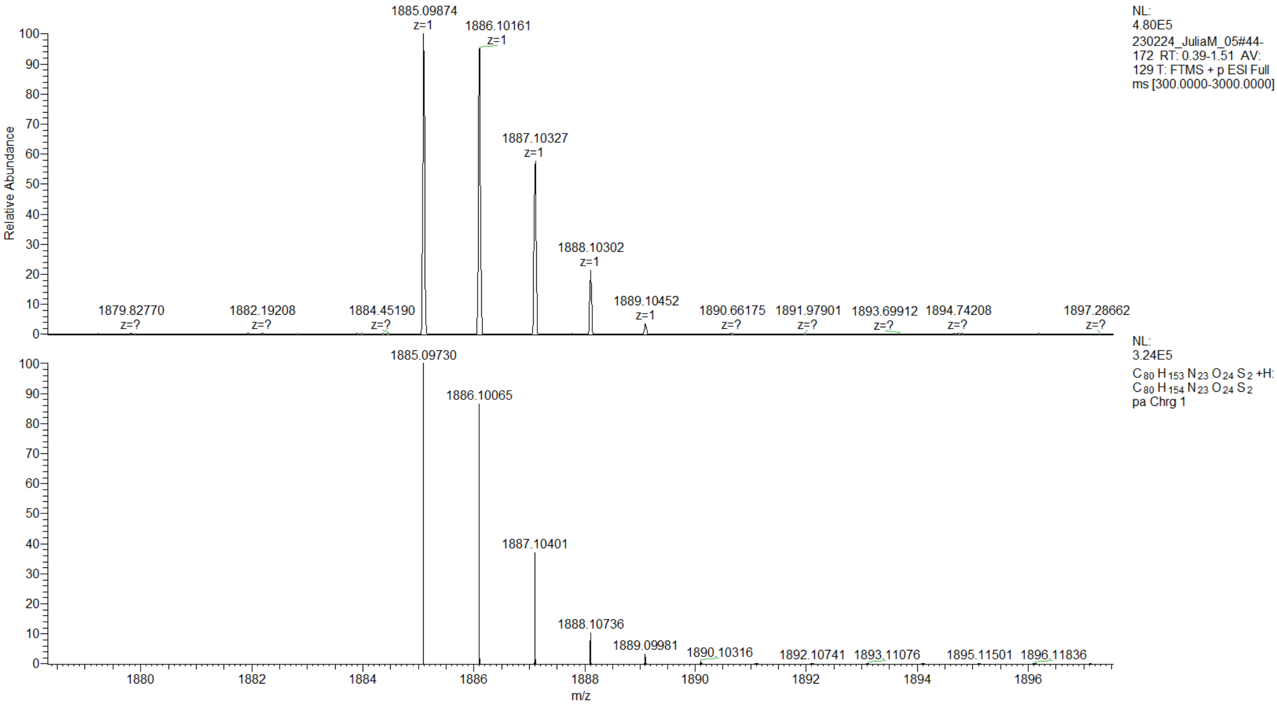

Figure S12. The RP-HPLC chromatogram after purification, calculated mass, chemical structures and high-resolution mass spectrum of NEO-*SS*-anoplin.

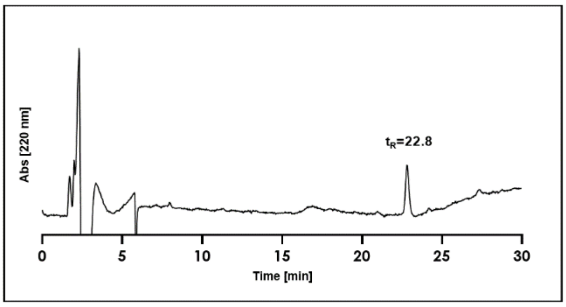

NEO-*SS*-anoplin[2-6]

M=1909.1 g/mol

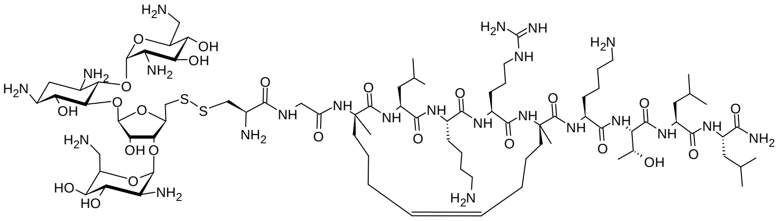

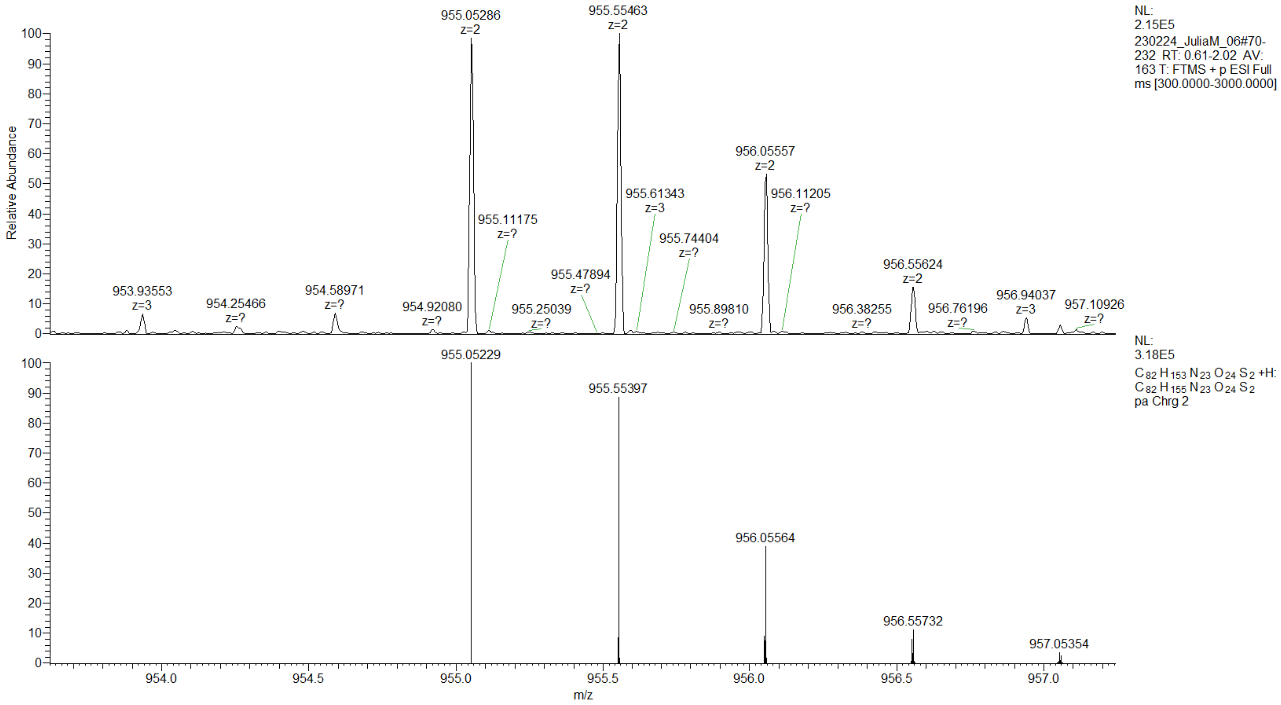

Figure S13. The RP-HPLC chromatogram after purification, calculated mass, chemical structures and high-resolution mass spectrum of NEO-*SS*-anoplin[2-6].

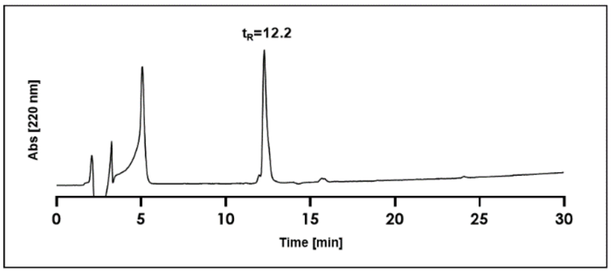

anoplin-*SS*-anoplin

M=2511.6 g/mol

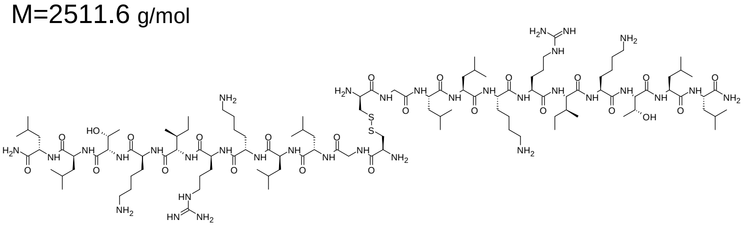

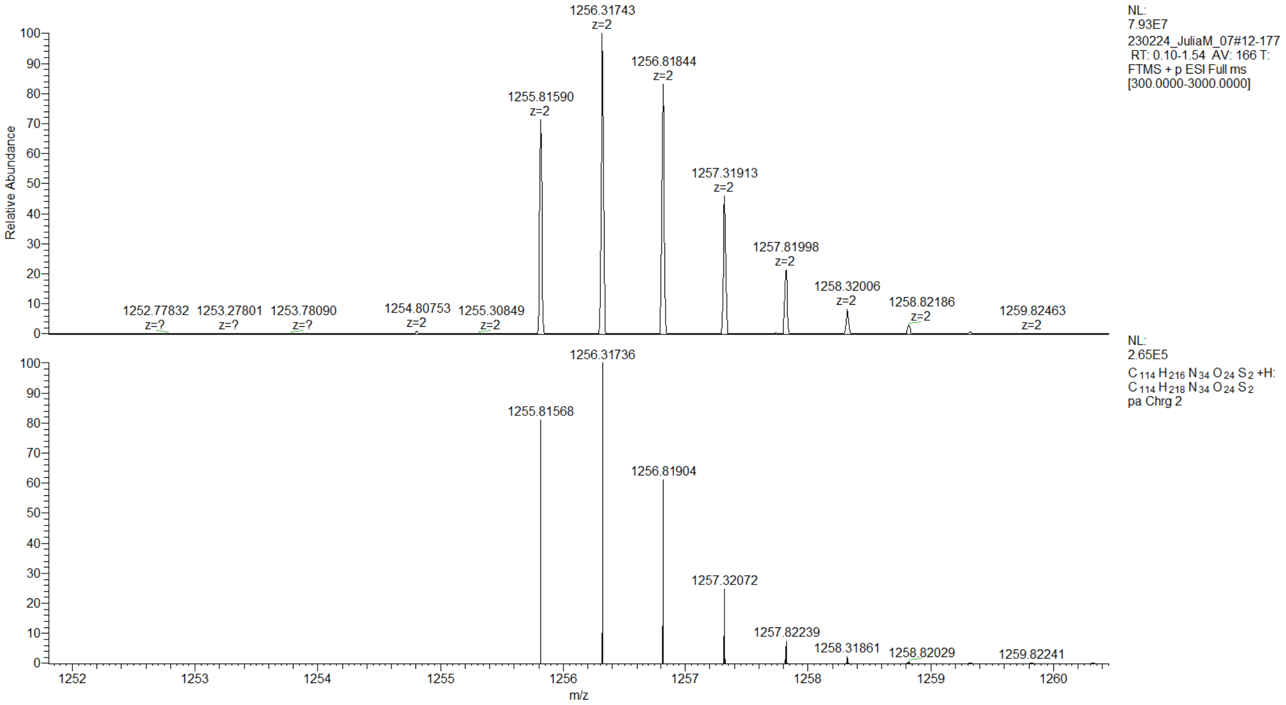

Figure S14. The RP-HPLC chromatogram after purification, calculated mass, chemical structures and high-resolution mass spectrum of anoplin-*SS*-anoplin.

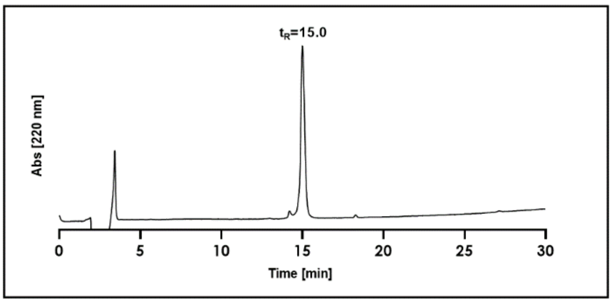

anoplin[2-6]-*SS*-anoplin[2-6]

M=2559.6 g/mol

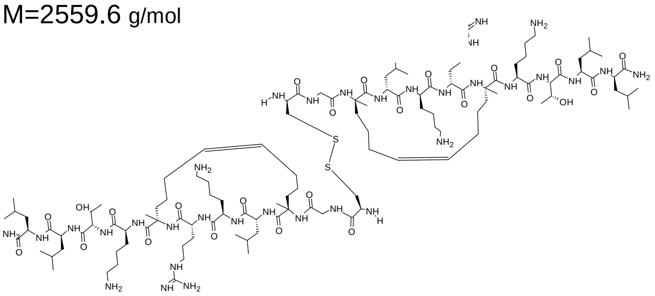

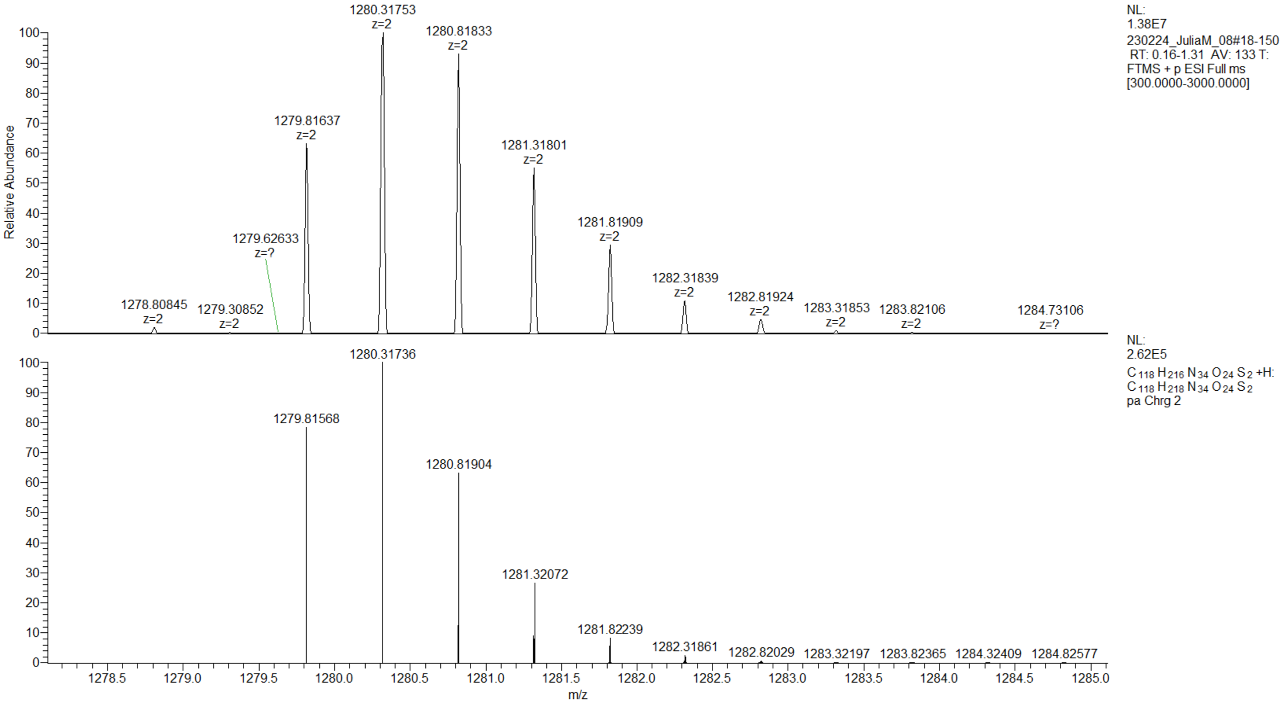

Figure S15. The RP-HPLC chromatogram after purification, calculated mass, chemical structures and high-resolution mass spectrum of anoplin[2-6]-*SS*-anoplin[2-6].

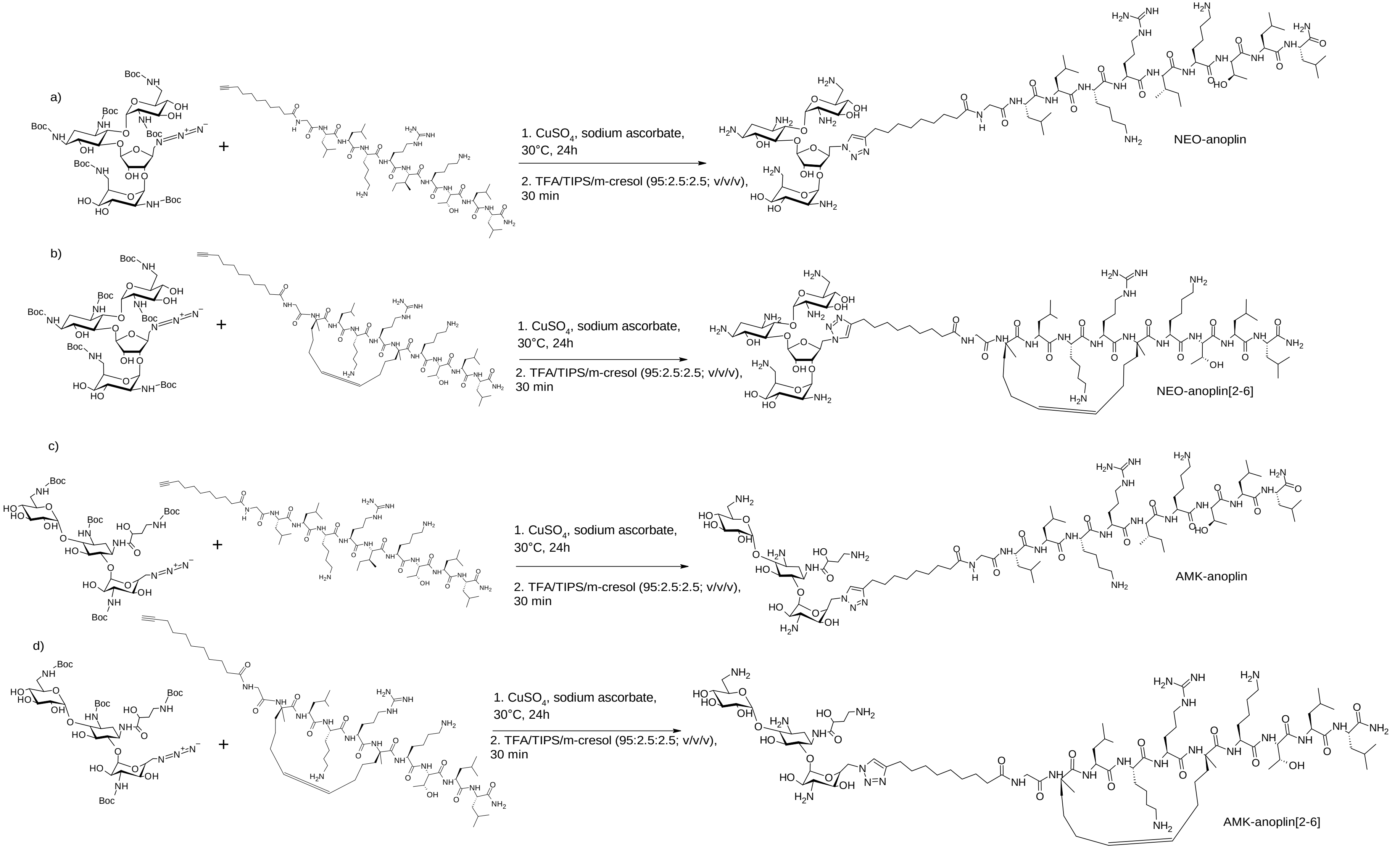

Scheme S1. Synthesis of the AMG-peptide conjugates: a) NEO-anoplin, b) NEO-anoplin[2-6], c) AMK-anoplin and d) AMK-anoplin[2-6] by CuAAC reaction.

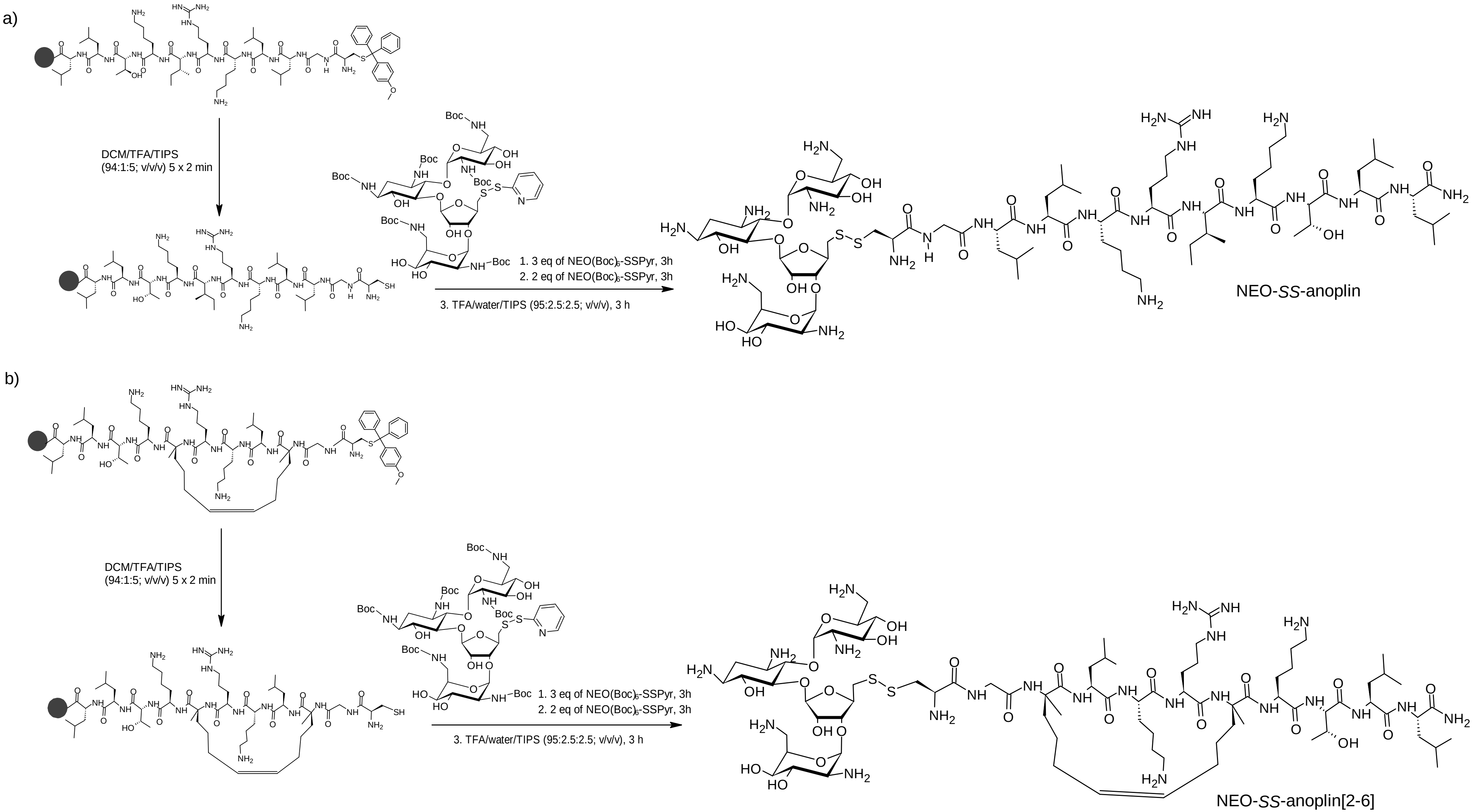

Scheme S2. Synthesis of the AMG-peptide conjugates a) NEO-*SS*-anoplin, b) NEO-*SS*-anoplin[2-6] by disulfide bond formation on the resin.

Table S1. Retention times, molecular masses and yields of the synthesized compounds.

Conjugates	Molecular mass [g/mol]		HPLC method[c]	t_R [min]	Yield[d] [%]
	Calculated[a]	Experimental[b]			
**NEO-anoplin**	1957.25321	1957.25587	25-70%/30 min	10.1	34.56
**NEO-anoplin[2-6]**	1981.25321	1981.25702	25-55%/30 min	13.7	49.89
**AMK-anoplin**	1928.2666	1928.23033	25-55%/30 min	13.7	45.12
**AMK-anoplin[2-6]**	1952.22666	1952.23110	0-55%/30 min	26.3	22.42
**NEO-*SS*-anoplin**	1885.09730	1885.09874	20-80%/30 min	8.5	5.60
**NEO-*SS*-anoplin[2-6]**	955.05229*	955.05286*	0-55%/30 min	22.6	3.31
**anoplin-*SS*-anoplin**	1255.81568*	1255.81590*	20-80%/30 min	12.2	ND
**anoplin[2-6]-*SS*-anoplin[2-6]**	1279.81568*	1279.81637*	20-80%/30 min	15.0	ND
**anoplin**	1153.81432	1153.1473	20-80%/30 min	11.4	ND
**anoplin[2-6]**	1177.81432	1177.81435	30-70%/30 min	10.3	ND

[a] The molecular mass of synthesized conjugates
[b] m/z (z=1) values obtained from high-resolution mass spectrometry (* z=2)
[c] The products were purified and analyzed by RP-HPLC in different gradient phases
[d] Yield calculated before desalinating the compound
ND – not determined

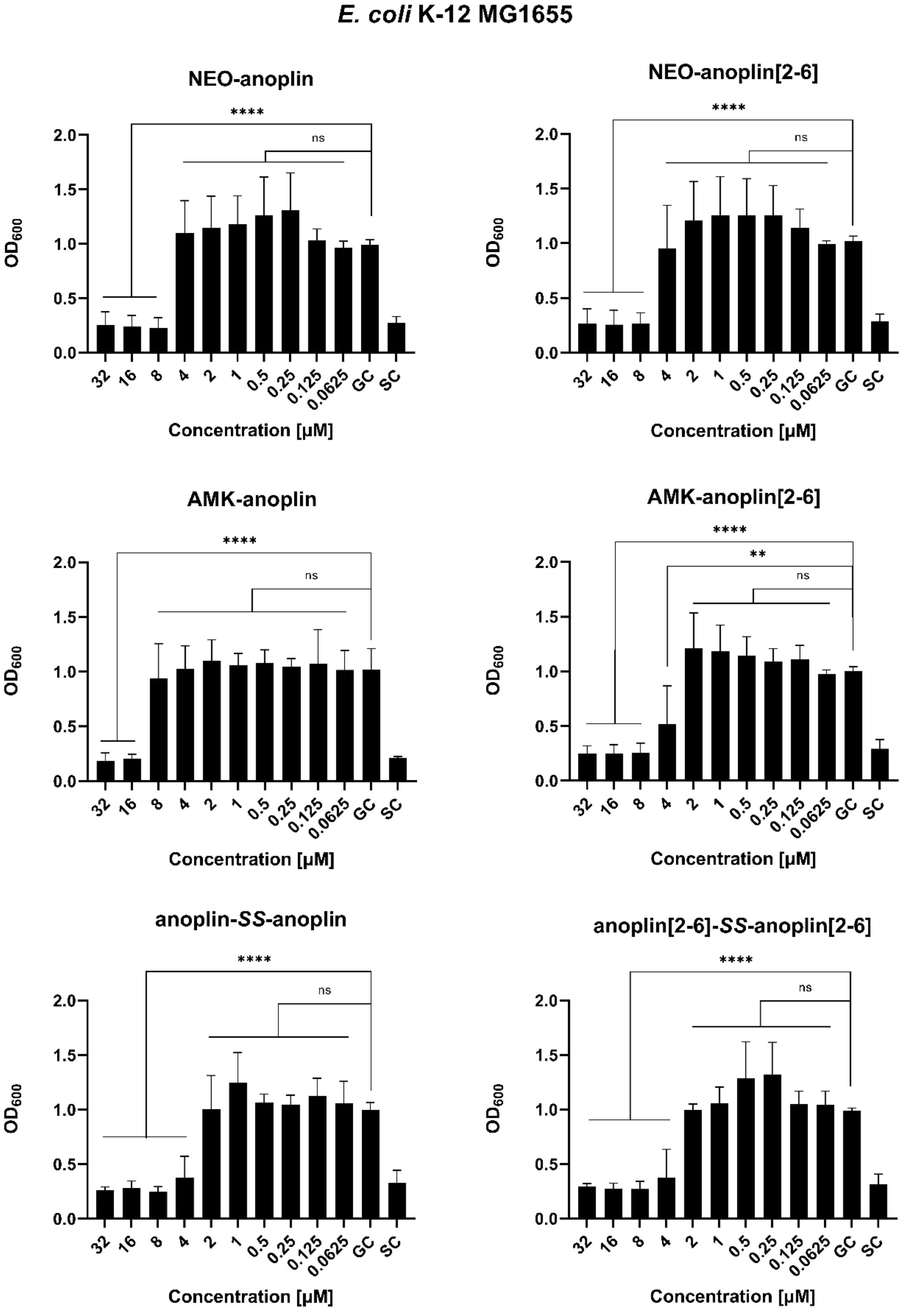

Figure S16. Optical density (OD_{600}) as a measure of *E. coli* K-12 MG1655 growth shown after 20 h incubation with various concentrations of: NEO-anoplin, NEO-anoplin[2-6], AMK-anoplin, AMK-anoplin[2-6], anoplin-*SS*-anoplin and anoplin[2-6]-*SS*-anoplin[2-6]. GC – growth control, SC – sterility control. Error bars represent the standard error of the mean; n=3. Statistical significance between the samples and GC: **** P < 0.0001, ** P < 0.01, ns – not significant.

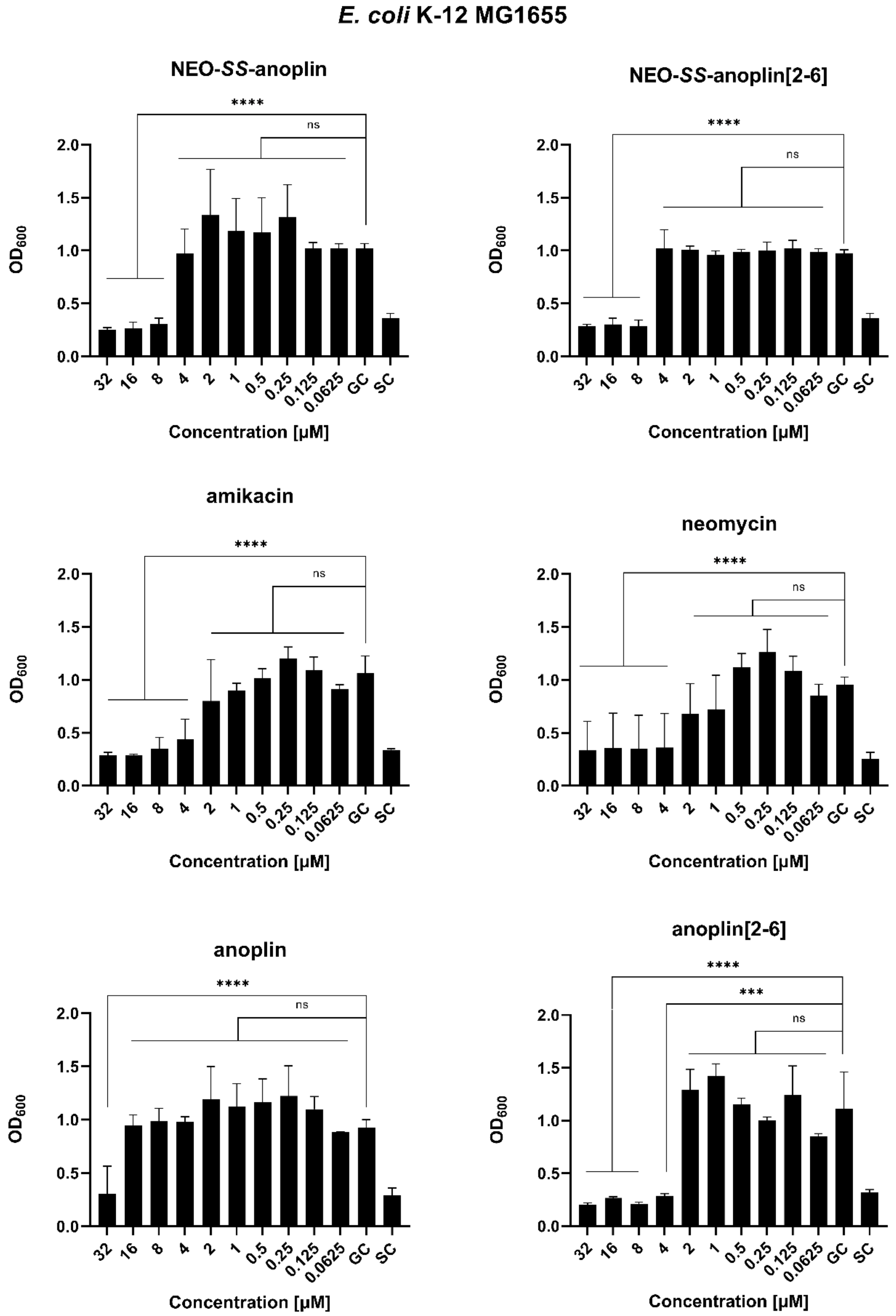

Figure S17. Optical density (OD_{600}) as a measure of *E. coli* K-12 MG1655 growth shown after 20 h incubation with various concentrations of: NEO-*SS*-anoplin, NEO-*SS*-anoplin[2-6], amikacin, neomycin, anoplin and anoplin[2-6]. GC – growth control, SC – sterility control. Error bars represent the standard error of the mean; n=3. Statistical significance between the samples and GC: **** P < 0.0001, *** P < 0.001, ns – not significant.

E. coli WR 3551/98

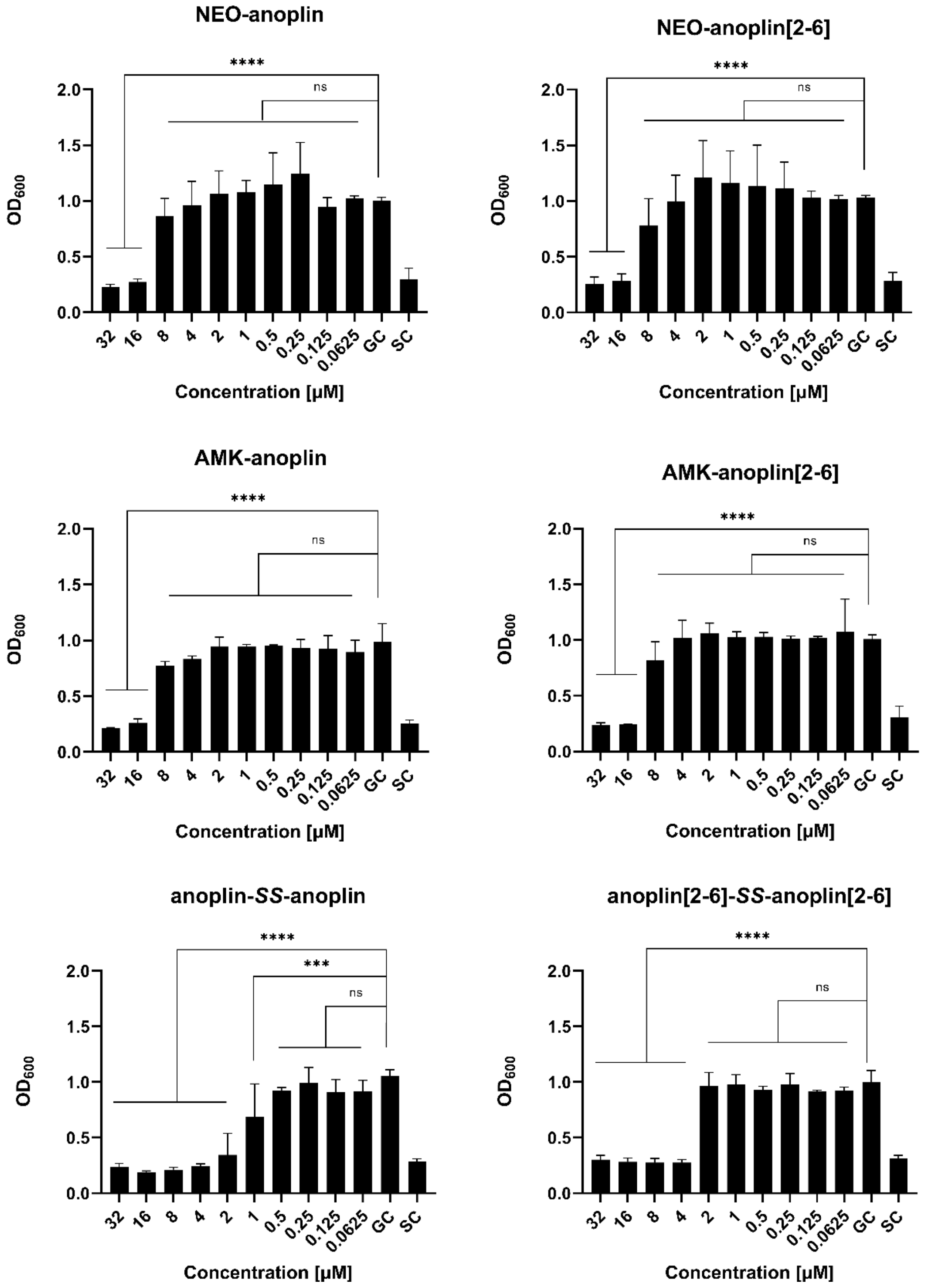

Figure S18. Optical density (OD_{600}) as a measure of *E. coli* WR 3551/98 growth shown after 20 h incubation with various concentrations of: NEO-anoplin, NEO-anoplin[2-6], AMK-anoplin, AMK-anoplin[2-6], anoplin-*SS*-anoplin and anoplin[2-6]-*SS*-anoplin[2-6]. GC – growth control, SC – sterility control. Error bars represent the standard error of the mean; n=3. Statistical significance between the samples and GC: **** $P < 0.0001$, *** $P < 0.001$, ns – not significant.

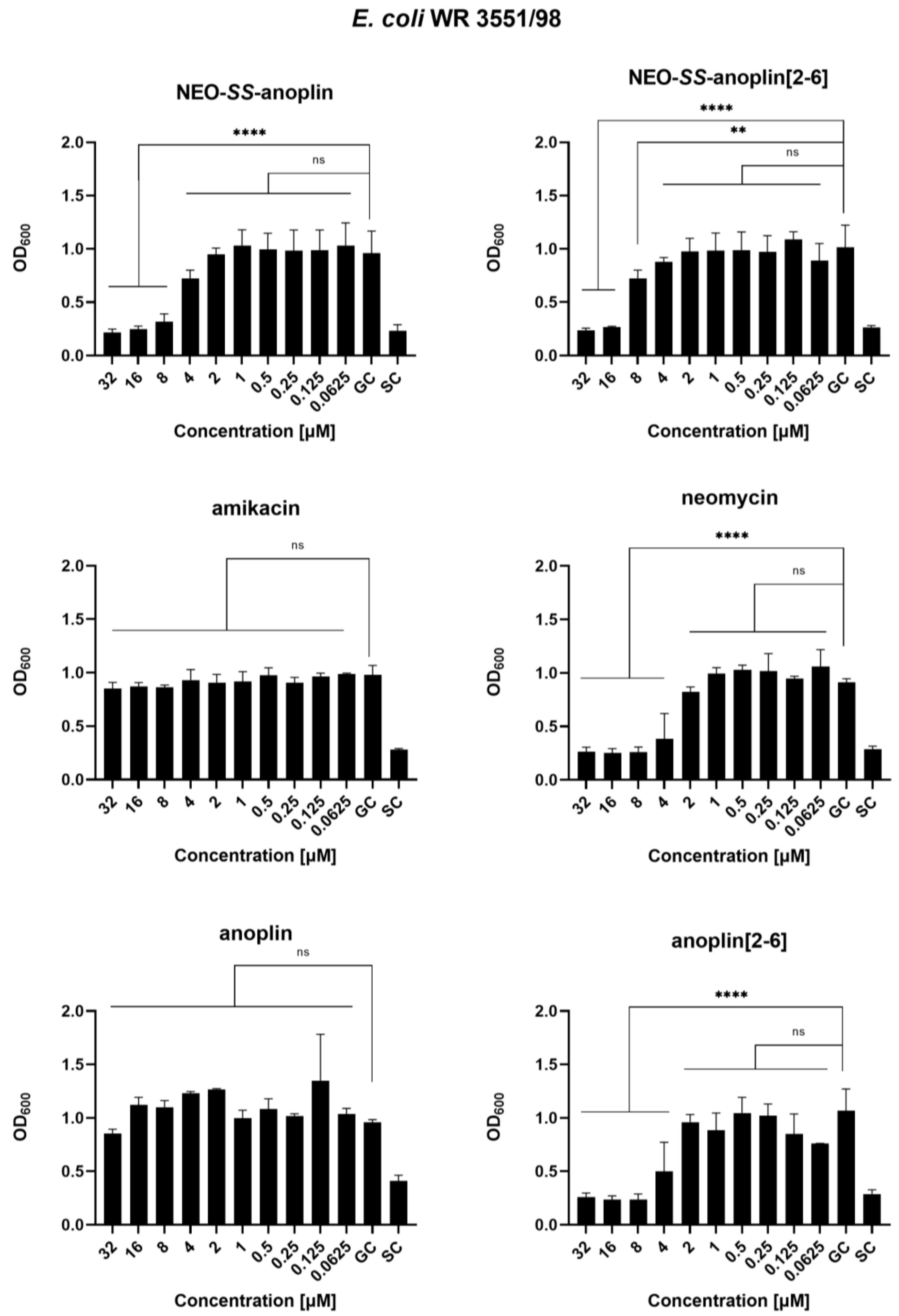

Figure S19. Optical density (OD_{600}) as a measure of *E. coli* WR3551/98 growth shown after 20 h incubation with various concentrations of: NEO-*SS*-anoplin, NEO-*SS*-anoplin[2-6], amikacin, neomycin, anoplin and anoplin[2-6]. GC – growth control, SC – sterility control. Error bars represent the standard error of the mean; n=3. Statistical significance between the samples and GC: **** P < 0.0001, ** P < 0.01, ns – not significant.

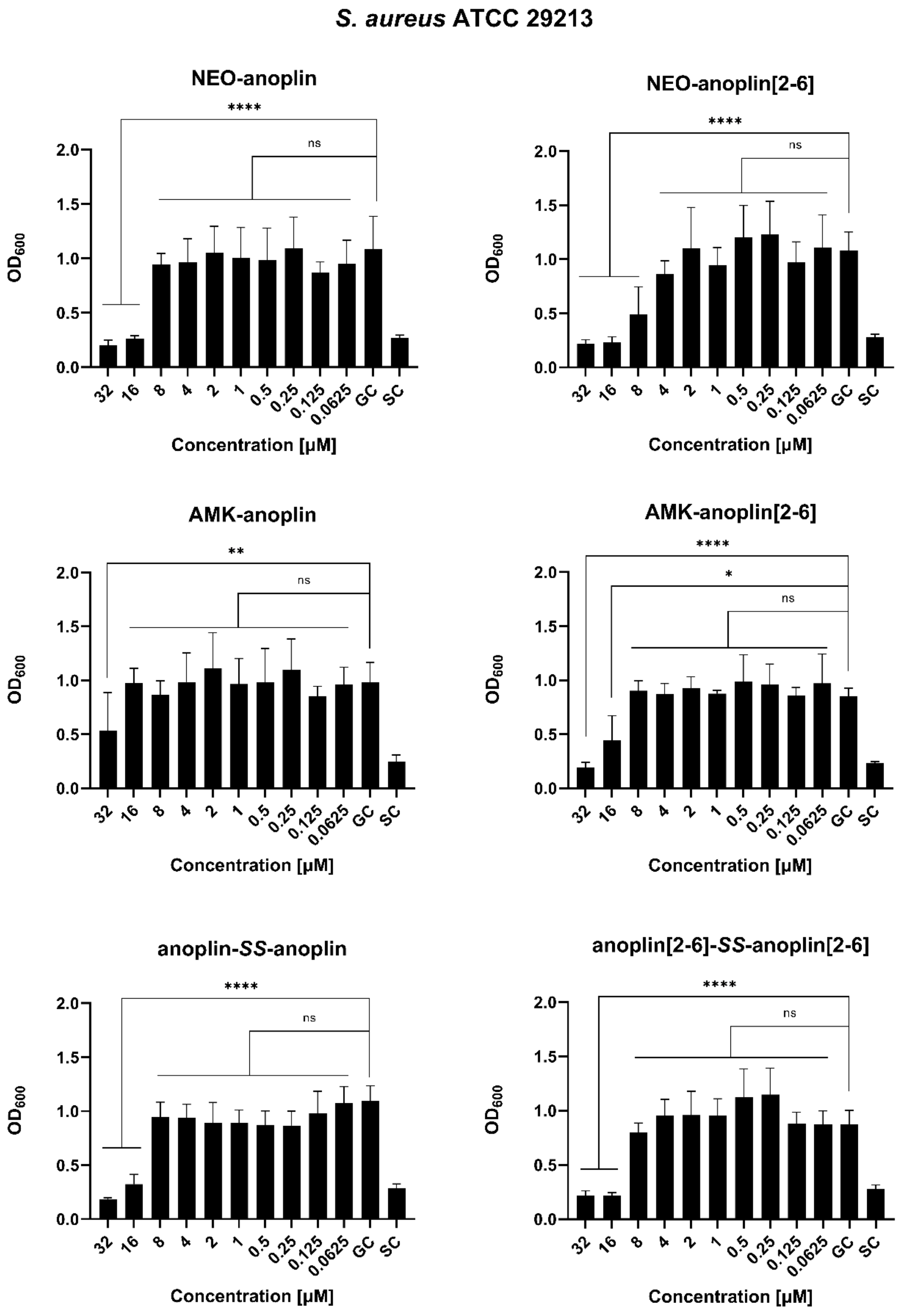

Figure S20. Optical density (OD_{600}) as a measure of *S. aureus* ATCC 29213 growth shown after 20 h incubation with various concentrations of: NEO-anoplin, NEO-anoplin[2-6], AMK-anoplin, AMK-anoplin[2-6], anoplin-*SS*-anoplin and anoplin[2-6]-*SS*-anoplin[2-6]. GC – growth control, SC – sterility control. Error bars represent the standard error of the mean; n=3. Statistical significance between the samples and GC: **** P < 0.0001, ** P < 0.01, * P < 0.05, ns – not significant.

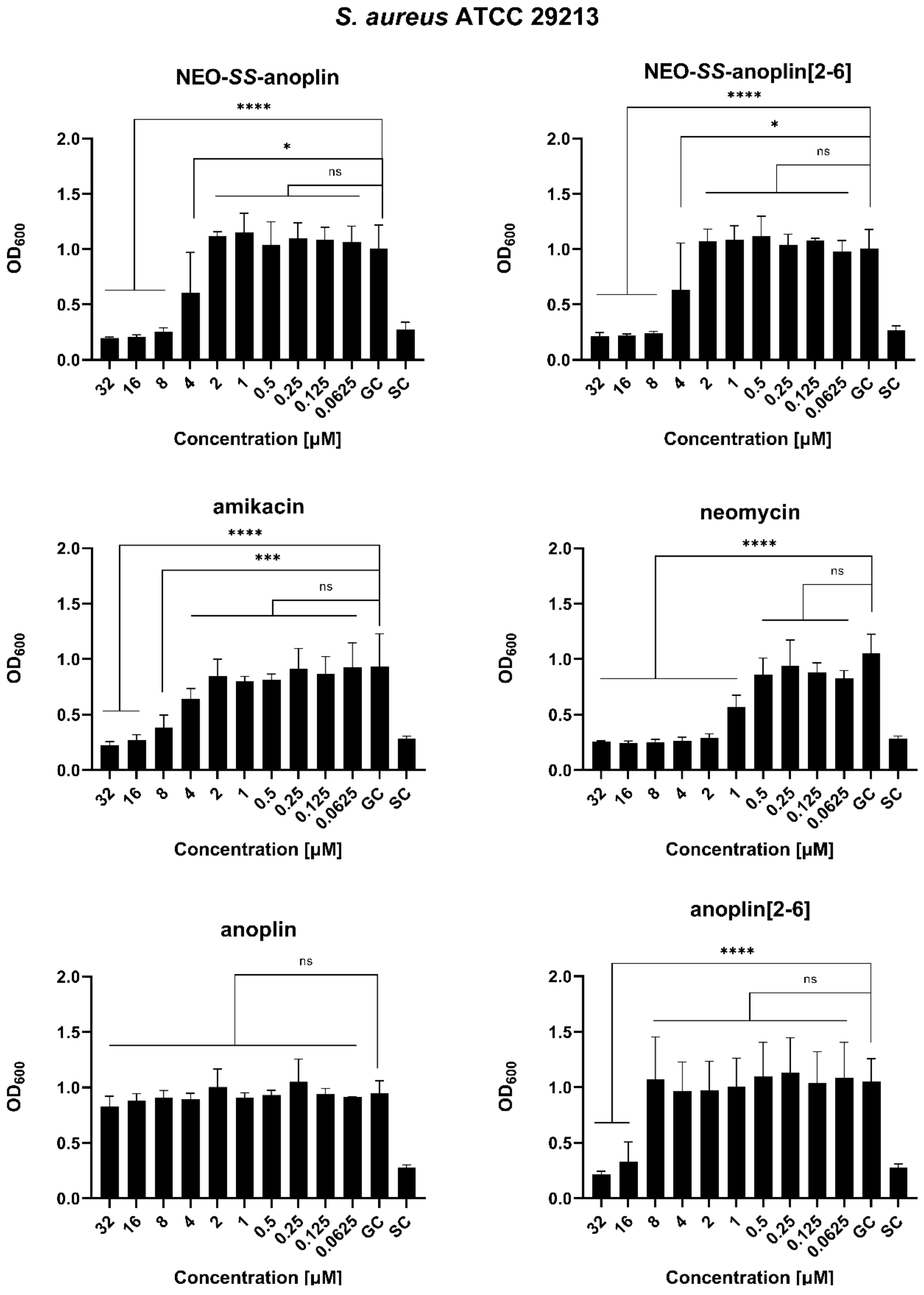

Figure S21. Optical density (OD_{600}) as a measure of *S. aureus* ATCC 29213 growth shown after 20 h incubation with various concentrations of: NEO-*SS*-anoplin, NEO-*SS*-anoplin[2-6], amikacin, neomycin, anoplin and anoplin[2-6]. GC – growth control, SC – sterility control. Error bars represent the standard error of the mean; n=3. Statistical significance between the samples and GC: **** P < 0.0001, *** P < 0.001, * P < 0.05, ns – not significant.

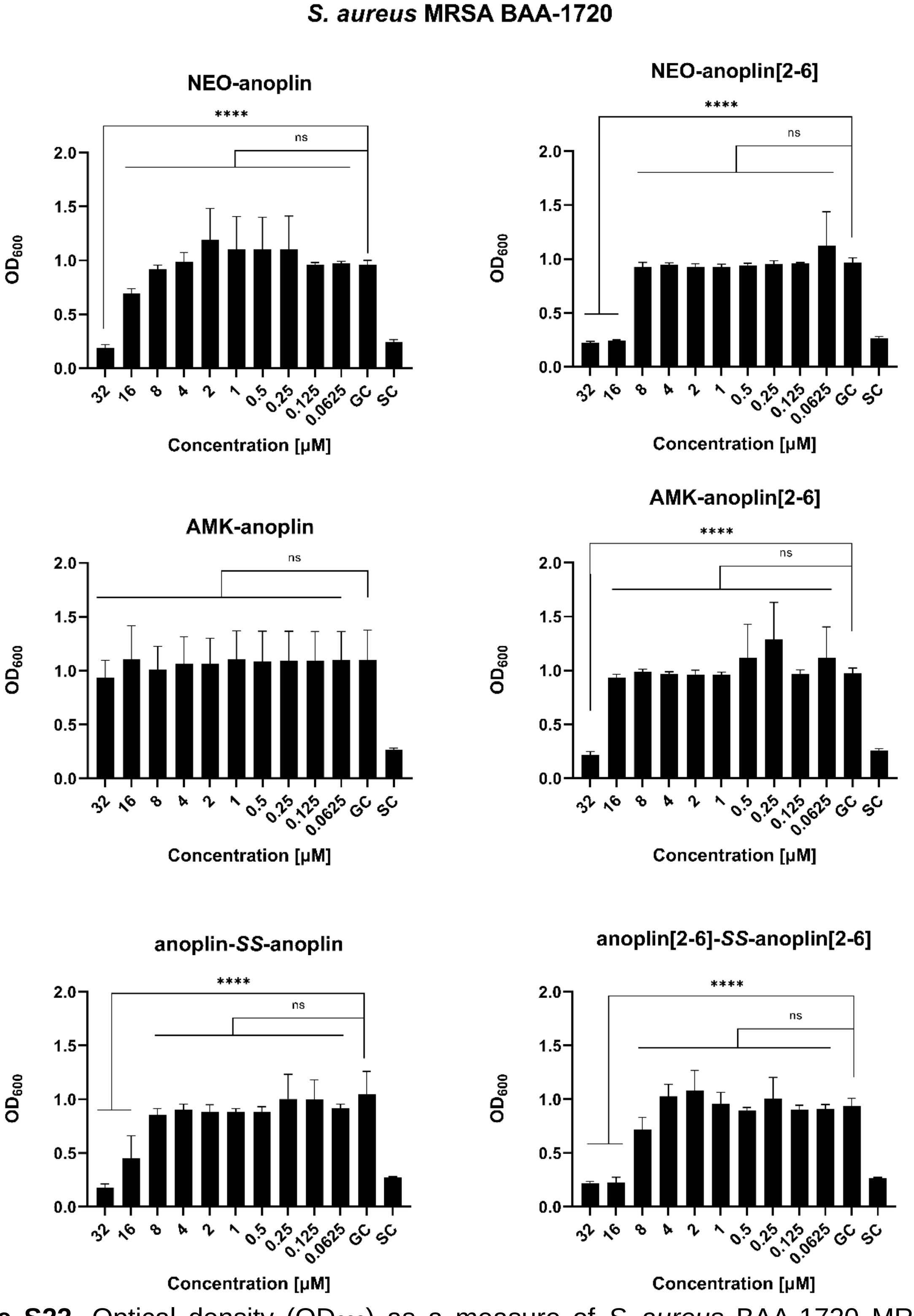

Figure S22. Optical density (OD_{600}) as a measure of *S. aureus* BAA-1720 MRSA growth shown after 20 h incubation with various concentrations of: NEO-anoplin, NEO-anoplin[2-6], AMK-anoplin, AMK-anoplin[2-6], anoplin-*SS*-anoplin and anoplin[2-6]-*SS*-anoplin[2-6]. GC – growth control, SC – sterility control. Error bars represent the standard error of the mean; n=3. Statistical significance between the samples and GC: **** P < 0.0001, ns – not significant.

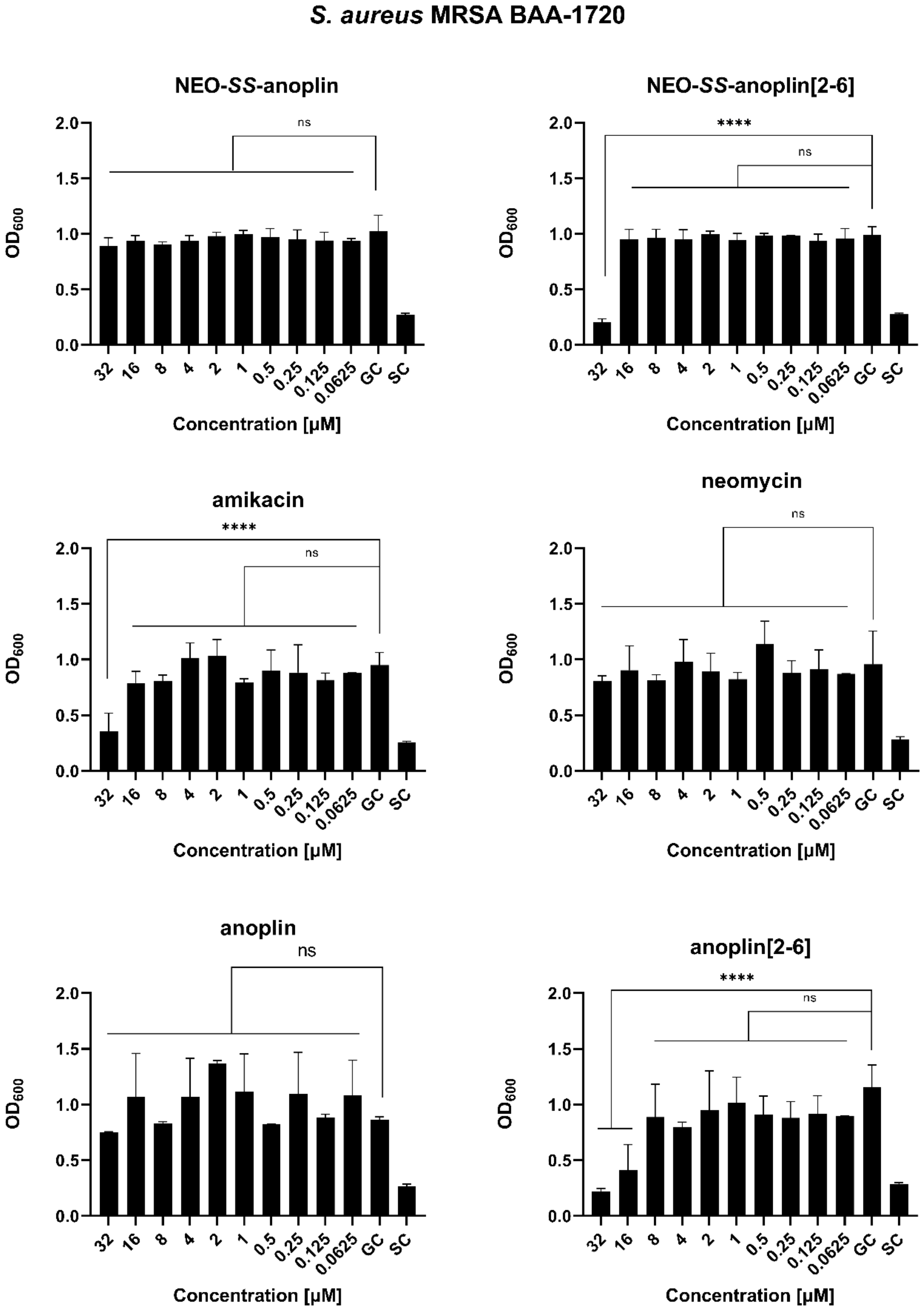

Figure S23. Optical density (OD_{600}) as a measure of *S. aureus* BAA-1720 MRSA growth shown after 20 h incubation with various concentrations of: NEO-*SS*-anoplin, NEO-*SS*-anoplin[2-6], amikacin, neomycin, anoplin and anoplin[2-6]. GC – growth control, SC – sterility control. Error bars represent the standard error of the mean; n=3. Statistical significance between the samples and GC: **** $P < 0.0001$, ns – not significant.

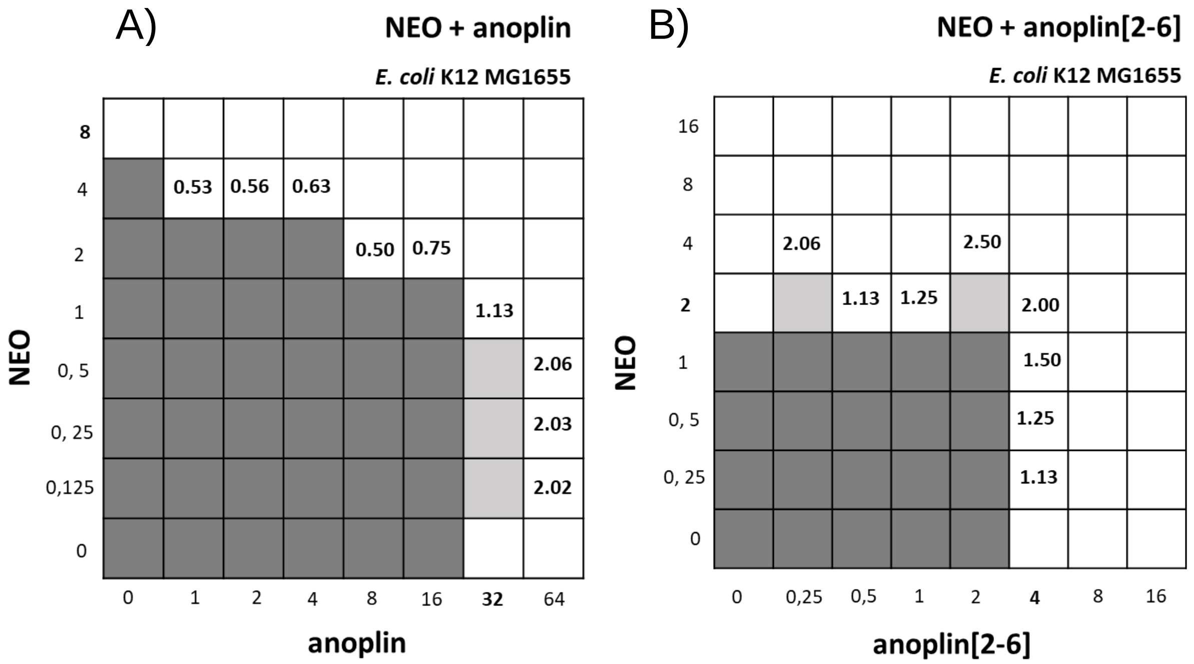

Figure S24. Typical checkerboard test combinations of A) NEO and anoplin and B) NEO and anoplin[2-6] determined for *E. coli* K12 MG1655 from one replicate. The calculated FIC index shows no synergistic effect but an additive interaction of the tested combination. White wells indicate no growth and dark grey indicate visible growth. Light grey indicates partial growth.

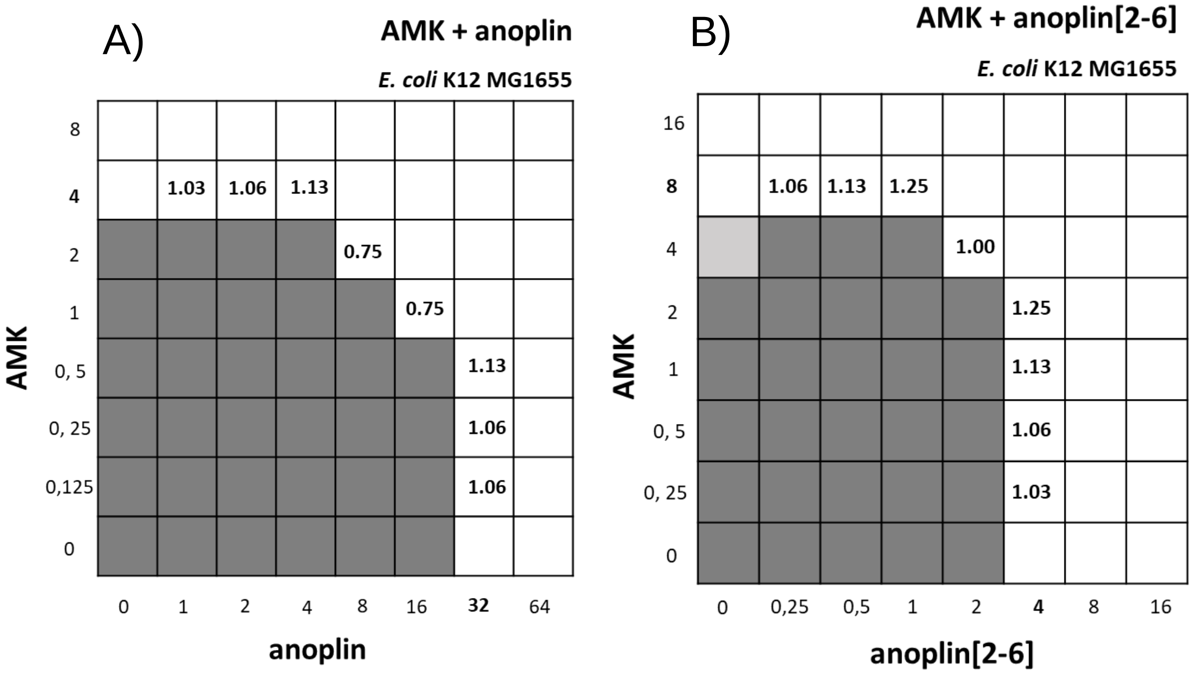

Figure S25. Typical checkerboard test combinations of A) AMK and anoplin and B) AMK and anoplin[2-6] determined for *E. coli* K12 MG1655 from one replicate. The calculated FIC index shows no synergistic effect but an additive interaction of the tested combination. White wells indicate no growth and dark grey indicate visible growth. Light grey indicates partial growth.

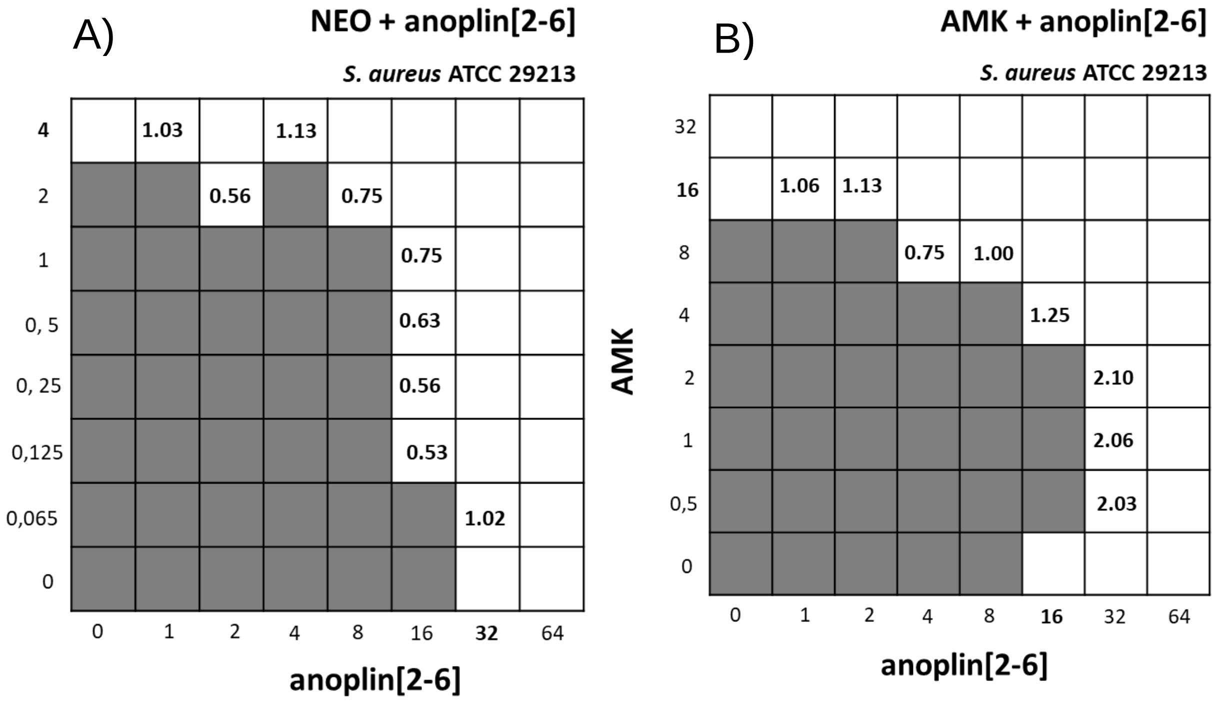

Figure S26. Typical checkerboard test combinations of A) NEO and anoplin[2-6] and B) AMK and anoplin[2-6] determined for *S. aureus* ATCC 29213 from one replicate. The calculated FIC index shows no synergistic effect but an additive interaction of the tested combination. White wells indicate no growth and dark grey indicate visible growth.
